# Phylogenomic analysis of carangimorph fishes reveals flatfish asymmetry arose in a blink of the evolutionary eye

**DOI:** 10.1186/s12862-016-0786-x

**Published:** 2016-10-21

**Authors:** Richard C. Harrington, Brant C. Faircloth, Ron I. Eytan, W. Leo Smith, Thomas J. Near, Michael E. Alfaro, Matt Friedman

**Affiliations:** 1 Department of Earth Sciences, University of Oxford, Oxford, OX1 3AN UK; 2 Department of Ecology & Evolutionary Biology and Peabody Museum of Natural History, Yale University, New Haven, CT 06520 USA; 3 Department of Biological Sciences and Museum of Natural Science, Louisiana State University, Baton Rouge, LA 70803 USA; 4 Department of Marine Biology, Texas A&M University at Galveston, Galveston, TX 77553 USA; 5 Biodiversity Institute and Department of Ecology and Evolutionary Biology, University of Kansas, Lawrence, KS 66045 USA; 6 Department of Ecology and Evolutionary Biology, University of California Los Angeles, Los Angeles, CA 90095 USA; 7 Museum of Paleontology and Department of Earth and Environmental Science, University of Michigan, 1109 Geddes Ave, Ann Arbor, MI 48109-1079 USA

**Keywords:** Adaptive radiation, Carangimorpha, Evolutionary innovation, Pleuronectiformes, UCE, Ultraconserved elements

## Abstract

**Background:**

Flatfish cranial asymmetry represents one of the most remarkable morphological innovations among vertebrates, and has fueled vigorous debate on the manner and rate at which strikingly divergent phenotypes evolve. A surprising result of many recent molecular phylogenetic studies is the lack of support for flatfish monophyly, where increasingly larger DNA datasets of up to 23 loci have either yielded a weakly supported flatfish clade or indicated the group is polyphyletic. Lack of resolution for flatfish relationships has been attributed to analytical limitations for dealing with processes such as nucleotide non-stationarity and incomplete lineage sorting (ILS). We tackle this phylogenetic problem using a sequence dataset comprising more than 1,000 ultraconserved DNA element (UCE) loci covering 45 carangimorphs, the broader clade containing flatfishes and several other specialized lineages such as remoras, billfishes, and archerfishes.

**Results:**

We present a phylogeny based on UCE loci that unequivocally supports flatfish monophyly and a single origin of asymmetry. We document similar levels of discordance among UCE loci as in previous, smaller molecular datasets. However, relationships among flatfishes and carangimorphs recovered from multilocus concatenated and species tree analyses of our data are robust to the analytical framework applied and size of data matrix used. By integrating the UCE data with a rich fossil record, we find that the most distinctive carangimorph bodyplans arose rapidly during the Paleogene (66.0–23.03 Ma). Flatfish asymmetry, for example, likely evolved over an interval of no more than 2.97 million years.

**Conclusions:**

The longstanding uncertainty in phylogenetic hypotheses for flatfishes and their carangimorph relatives highlights the limitations of smaller molecular datasets when applied to successive, rapid divergences. Here, we recovered significant support for flatfish monophyly and relationships among carangimorphs through analysis of over 1,000 UCE loci. The resulting time-calibrated phylogeny points to phenotypic divergence early within carangimorph history that broadly matches with the predictions of adaptive models of lineage diversification.

**Electronic supplementary material:**

The online version of this article (doi:10.1186/s12862-016-0786-x) contains supplementary material, which is available to authorized users.

## Background

During the past decade, a series of molecular phylogenetic analyses drawing on increasingly larger samples of taxa and genetic loci have transformed our understanding of evolutionary relationships among acanthomorphs or spiny-rayed fishes [1–6], a hyperdiverse lineage that includes nearly one in three living vertebrate species. These studies support the monophyly of many clades previously recognized by morphological phylogeneticists (e.g., Tetraodontiformes, Lophiiformes), but reject the coherence of other classical groups (e.g., Scombroidei inclusive of billfishes [7], Labroidei [8]) by removing some of their core members to other, distantly related lineages [9–11]. In resolving the ‘bush’ at the top of the teleost tree of life, these molecular phylogenies have exposed striking examples of morphological, physiological, and functional convergence among acanthomorphs [10, 11], and revealed unexpected groupings of lineages not previously regarded as closely related [11].

A well-supported radiation [1, 12, 13], variously termed Clade L [14], Carangimorpha [2, 3], or Carangimorpharia [4, 15], represents one of the most surprising features of the emerging picture of acanthomorph interrelationships. Carangimorphs include anatomically disparate lineages characterized by remarkable behavioral and anatomical novelties: eye and brain heating organs coupled with long rostra and numerous specializations for rapid swimming in Xiphioidei (marlins and swordfishes) [16]; cranial adhesion discs and commensal lifestyle in Echeneidae (remoras) [17, 18]; expanded, tactile pectoral-fin rays in Polynemidae (threadfins) [19]; sophisticated spine-based venom delivery systems in Carangidae (scombroidin jacks) [20]; and use of water jets to capture aerial prey in Toxotidae (archerfishes) [21]. These specializations are joined by even more singular innovations in arguably the most peculiar carangimorph lineage: Pleuronectiformes (flatfishes). Flatfishes, including familiar food fish like halibut, sole, and plaice, are characterized by profound cranial asymmetry resulting from the migration of one eye to the opposite side of the skull during larval metamorphosis. This extreme developmental resculpturing of the head permits adult flatfishes to rest on the seafloor on their eyeless or ‘blind’ side, leaving both eyes of the ‘eyed’ side unobstructed by sediment.

**Fig. 1:**
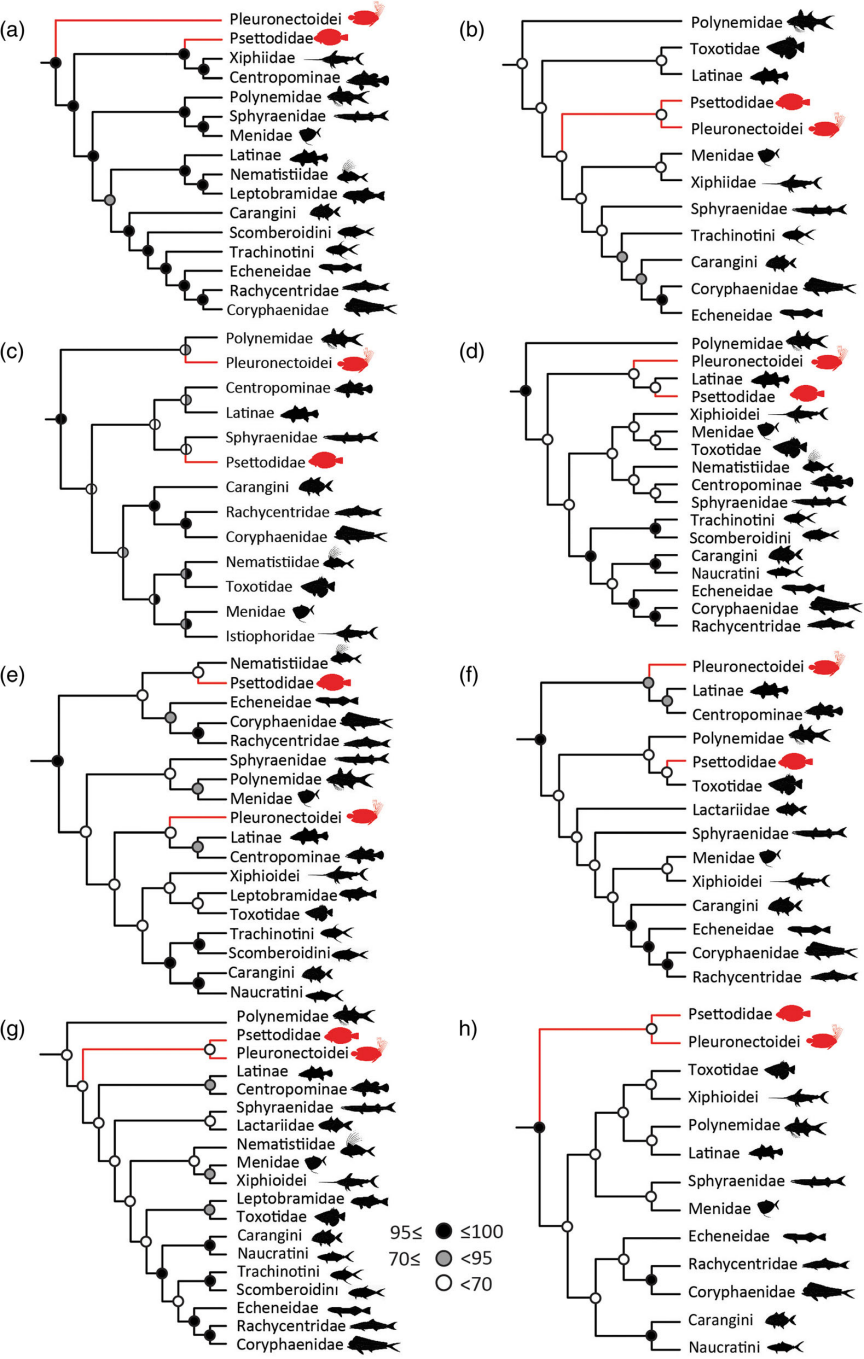
Previous molecular phylogenies of Carangimorpha, emphasizing the relationships of flatfishes. **a** Smith & Wheeler [1] (5 loci); **b** Li et al. [2] (4 loci); **c** Li et al. [78] (13 loci); **d** Near et al. [3] (10 loci); **e** Betancur-R. et al. [4] (21 loci); **f** Campbell et al. [38] (6 loci); **g** Betancur-R. & Ortí [34] (23 loci); **h** Campbell et al. [39]. Flatfishes are indicated in red. Discs indicate nodal support as assessed by bootstrapping. Bayesian posterior probabilities (x 100) are indicated by the right-hand side of discs in (**h**)

Although carangimorph monophyly is well supported, relationships among its principal lineages are weakly supported and highly variable between studies (Fig. 1). Flatfishes provide the most concrete illustration of the uncertainty in carangimorph relationships. Ichthyologists have overwhelmingly regarded the orbital migration and associated lateralized behavior of flatfishes as unique innovations that strongly support pleuronectiform mononphyly [22–30] (but see [31–33]). However, analyses that do recover a flatfish clade provide only weak statistical support for its monophyly (bootstrap support < 70 %; Fig. 1) [2, 4, 34–36]. Several published phylogenies support the monophyly of a subset of flatfishes representing Pleuronectoidei of anatomical classifications [23, 37, 38] and place *Psettodes* as the sister taxon of a closely related—but symmetrical—lineage that varies among analyses (Fig. 1; Centropomidae [snooks] + Xiphoidei [1]; Sphyraenidae [barracudas] [2]; Latinae [Nile perches] [3]; Nematistiidae [roosterfish] [4]; Toxotidae [38]). Although trees favoring a polyphyletic origin of Pleuronectiformes lack sufficient statistical support to reject the morphological null hypothesis of monophyly [34], some researchers have nevertheless concluded that asymmetry arose independently in *Psettodes* and pleuronectoids [38, 39]. These conclusions resurrect a pre-cladistic hypothesis in which generalized percomorph traits apparent in *Psettodes* suggest that cranial symmetry evolved within this lineage independently of that in pleuronectoids [31, 32], an inference that casts uncertainty on the phylogenetic placement and evolutionary significance of early flatfishes showing incomplete orbital migration [39].

The recent debate regarding flatfish monophyly spotlights the difficulties faced by many phylogenetic studies, particularly in the use of molecular data for radiations characterized by short internodes deep in evolutionary time. Methodological challenges, such as accounting for base compositional bias (i.e., non-stationarity [40]) and long branch attraction [41, 42], as well as natural phenomena such as horizontal gene flow and incomplete lineage sorting (ILS) can result in inference of gene trees that do not reflect a clade’s history of speciation [43–45]. Even under certain scenarios of branch length in species trees, the most frequent gene trees do not reflect the topology of the species tree (the so-called anomaly zone [46]). The use of analytical approaches to account for non-stationarity or application of the coalescent model for ILS is important for improving accuracy of gene tree and species tree estimation, but the addition of large numbers of unlinked loci may be the most direct approach for improving phylogenetic accuracy in the face of these processes [47]. Thus far, incrementally larger DNA sequence datasets of up to 23 loci have produced inconsistent increases in support for relationships among major carangimorph lineages, particularly with regard to flatfish monophyly (Fig. 1) and the identification of a symmetrical sister group.

Using recent advances in phylogenomics and high-throughput sequencing, we assembled a dataset of ultraconserved DNA elements (UCEs) and their flanking sequences representing over 1000 loci sampled from 45 carangimorph species. Here we use this novel dataset in conjunction with the rich fossil record of Carangimorpha to: (i) establish a well-supported hypothesis of relationships among anatomically disparate carangimorph lineages; (ii) provide a statistically decisive molecular solution to the ‘pleuronectiform problem’; and (iii) estimate divergence times for carangimorphs, with an emphasis on constraining the timescale over which remarkable anatomical innovations such as flatfish asymmetry and other specialized carangimorph bodyplans likely arose.

## Methods

We use a probe set developed for application to acanthomorph phylogenetics to generate sequence data for approximately 1200 UCE loci [48]. These loci vary in size and number of variable sites per locus, but average between 300 and 500 nucleotides in length. This approach has provided resolution across a range of evolutionary depths in phylogenetic studies of acanthomorphs [5]. Gilbert et al. [49] examined the phylogenetic informativeness of a subset of the probe set used in this analysis, and found that it provides greater informativeness across ages during which carangimorphs diversified than do protein-coding genes previously used in phylogenetic analysis of acanthomorphs (including carangimorphs). Information on DNA isolation, library preparation, sequencing, and data pipelining is provided in Additional file 1: Methods 1. Raw read data are archived in the NCBI Sequence Read Archive (SRA) under accession numbers SAMN05784507, SAMN05786321-SAMN05785372, SAMN05919513, SAMN05919514. Our high-throughput sequencing generated uneven coverage across taxa and loci, resulting in an incomplete data matrix for all 1200 UCE loci. While increasing the number of nucleotides and loci is desirable for phylogenetic analysis, empirical studies of the impact of missing data on resolving difficult nodes show diminishing returns at varying thresholds of incompleteness (e.g., 25 % incomplete in [50] and 50 % incomplete in [51]). In order to evaluate the role of missing data in our matrices and whether tree topology and clade support values varied with the addition of more, but sparsely sampled loci, we performed phylogenetic analyses on 75 %, 95 %, and 100 % complete alignment matrices.

### Concatenated analyses of UCE data

Across all data matrices, we conducted 20 maximum-likelihood (ML) searches for the phylogenetic tree that best fit the data using the best-fitting partitioning scheme, RAxML v. 8.0.19 [52], and the GTRGAMMA model. The best-fitting partitioning scheme was obtained using the Bayesian Information Criterion and hcluster search in PartitionFinder v 1.1.1 [53, 54], applying equal weights for overall rates, base frequencies, model parameters, and the alpha parameter. We rooted the tree on the lanternfish *Ceratoscopelus warmingii*. Following the best tree search, we used RAxML to generate non-parametric bootstrap pseudoreplicates using the autoMRE function of RAxML, and reconciled the best fitting ML tree with the bootstrap replicates. We also performed Bayesian analyses of each concatenated data set using ExaBayes [55]. We input the concatenated supermatrix and best-fitting partitioning scheme to ExaBayes and ran four independent analyses of 5 × 10^5^ to 1 × 10^6^ iterations. We checked results for convergence by ensuring the average standard deviation of split frequencies (ASDSF) was <1 %, effective sample size (ESS) values were >200, and the potential scale reduction factor (PSRF) for estimated parameters was approximately 1.0. We also visualized parameter estimates and ESS values in Tracer v 1.6 [56]. We generated the 50 % credible set of trees from the posterior distribution of possible topologies using the consense program from ExaBayes (burn-in: 25 %; thinning: 500).

### Gene tree-species tree analysis of UCE data

We inferred gene trees from the individual locus alignments that comprised the 75 % complete matrix described above, and we subset those gene trees based on the names of loci present in the 95 % and 100 % complete data matrices for gene tree-species tree analysis of each dataset, respectively. To infer locus-specific trees from each data matrix, we conducted 20 maximum-likelihood (ML) searches for the phylogenetic tree that best fit the data using RAxML v. 8.0.19 [52] and the GTRGAMMA model. We also used RAxML to generate ~100 non-parametric bootstrap pseudoreplicates from each locus. We inferred the species tree from individual gene trees using ASTRAL v. 4.4.4 [57] under the bootstrap option. All other ASTRAL parameters were the defaults.

### Analysis of concordance among loci

Unlinked loci exhibit independent histories of sorting through populations over evolutionary timescales, and this can result in gene trees with non-matching topologies. Therefore, calculations of statistical support for clades derived from total-evidence concatenation or species tree analyses may not accurately demonstrate the genome-wide agreement on a lineage’s evolutionary history [58]. To evaluate the level of concordance among loci, and in particular the number of loci that recover a flatfish clade, we performed Bayesian Concordance Analysis (BCA) using BUCKy version 1.4.4 [59, 60]. BUCKy uses posterior tree density distributions from individual loci to estimate the proportion of loci that support branching patterns in trees (i.e., concordance factors, CF), and assembles taxa with highest CF values into clades with the least conflict in order to generate a primary concordance tree. BUCKy also applies concordance factors in a quartet-joining algorithm to construct a population tree, which is consistent with a coalescent framework species tree when gene tree discordance is due to ILS [61].

We generated gene tree distributions for each UCE locus using MrBayes version 3.2.6 [62], with MCMC chains run for 1 million generations using HKY model, sampling trees every 200 generations, and discarding the first 2500 as burnin. With these gene tree distributions as input, we used BUCKy to estimate the proportion of gene trees supporting possible relationships among taxa, and to generate a primary concordance and population trees. BUCKy analyses were run for 1 million generations on 4 chains, and a burnin of 100,000 generations. We performed analyses over a range of the discordance prior values (alpha values of: 0.1, 1.0, 10, and 100). Because sequencing was uneven across taxa and loci, we aimed to maximize the number of loci that informed CF estimation by using a subset of taxa, with one representative from each major lineage, including *Psettodes* and two other flatfish (*Psettodes erumei*, *Cyclopsetta fimbriata*, *Citharoides macrolepis*, *Lates calcarifer*, *Centropomus medius*, *Toxotes jaculatrix*, *Leptobrama muelleri*, *Xiphias gladius*, *Echeneis naucratoides*, *Coryphaena hippurus*, *Trachinotus blochii*, *Alepes kleinii*, *Mene maculatus*, *Sphyraena sphyraena*, and *Polydactylus sexfilis*).

### Divergence time estimation

We estimated divergence times of carangimorph lineages using both a relaxed molecular clock approach implemented in BEAST v 1.8 [63] and an approximate likelihood calculation in PAML [64]. Due to computational limitations in *BEAST*, we performed a series of four replicate analyses using different sets of 75 randomly selected loci from the 95 % complete matrix (596 loci). For each set of 75 loci, we used PartitionFinder [53] to identify optimal partitioning schemes and assign loci to partitions. This produced between 5 and 8 partitions for each dataset. In BEAST, we applied an uncorrelated lognormal clock model (UCLN), GTRGAMMA site substitution model, and a birth-death speciation tree model. We constrained the topology to match that of the Bayesian and maximum likelihood analyses of the partitioned, concatenated 596 loci analyses, and incorporated age priors for 16 nodes on the carangimorph phylogeny. We based minima on fossil occurrences, and we specified lognormal prior distributions empirically informed by the mean and 95 % upper bounds estimated by the age of first appearance of successive outgroups [65]. We provide details of these age priors and the fossil data upon which they are based in Additional file 2. To assess convergence on divergence date estimates, we ran 5 analyses for each set of 75 randomly selected loci for 200 million generations. We used Tracer v 1.6 [56] to assess the convergence of model parameters and ensure lack of directional trends in trace plots of parameters and adequate mixing of the MCMC chain (ensuring that ESS was greater than 200). We set burn-in at 20 million generations, combined individual analyses for each of the individual 5 sets of analyses respectively, using LogCombiner v 1.8 [63], and constructed a maximum clade credibility consensus tree using TreeAnnotator v 1.8 [63].

We also estimated divergence times with the MCMCTree package of PAML version 4.8a [64] using likelihood approximation and an independent clock model for the 596 UCE loci dataset. As with the BEAST analysis, we constrained the topology to match that of the Bayesian and maximum likelihood analyses of the partitioned, concatenated 596 loci analyses. Calibration priors were applied to the same nodes as in BEAST analyses, using soft upper (95 %) and lower (1e-300) bounds. To arrive at a partitioning scheme that accounted for variation across sites but that was simple enough to be implemented in PAML, we used APE [66] to root the ExaBayes tree inferred from the 95 % complete matrix, dropped the tip representing the outgroup lineage (*Ceratoscopelus warmingii*), and set the branch lengths of the resulting tree to 1.0. We then removed *Ceratoscopelus warmingii* from the concatenated, 95 % complete supermatrix, and we input the supermatrix and guide tree to DendroPy [67]. We used the *parsimony_score* method in DendroPy to calculate the number of parsimony informative changes in each site pattern. We used kmeans clustering in R to cluster the distribution of parsimony scores by plotting the within-group sum of squares of parsimony score by a number of potential clusters (range 1–15). Visual examination of the resulting plot suggested five was the appropriate number of clusters. We then clustered the data, computed the cluster means, and assigned individual sites to one of the five clusters. We used a new program in PHYLUCE (phyluce_align_get_aligns_partitioned_by_cluster) to create a partitioned, concatenated alignment where each partition represented one of the five clusters, and we assigned an HKY85 model to each partition. We used the following values for other PAML options and model parameters: BDparas: 1, 1, 0; kappa_gamma: 6, 2; alpha_gamma: 1, 1; rgene_gamma: 2, 203.72, 1; sigma2_gamma: 2, 5, 1. We conducted 8 replicate MCMCTree runs sampling every 250 generations after 5000 generations of burnin for a total of 10000 samples per run. Tracer v 1.6 [56] was used to visually assess convergence and calculate the ESS for all model parameters. Once satisfactory ESS values were attained and convergence verified by visual inspection using Tracer, we combined log files from independent runs to calculate final posterior distributions for all parameters.

## Results

**Fig. 2:**
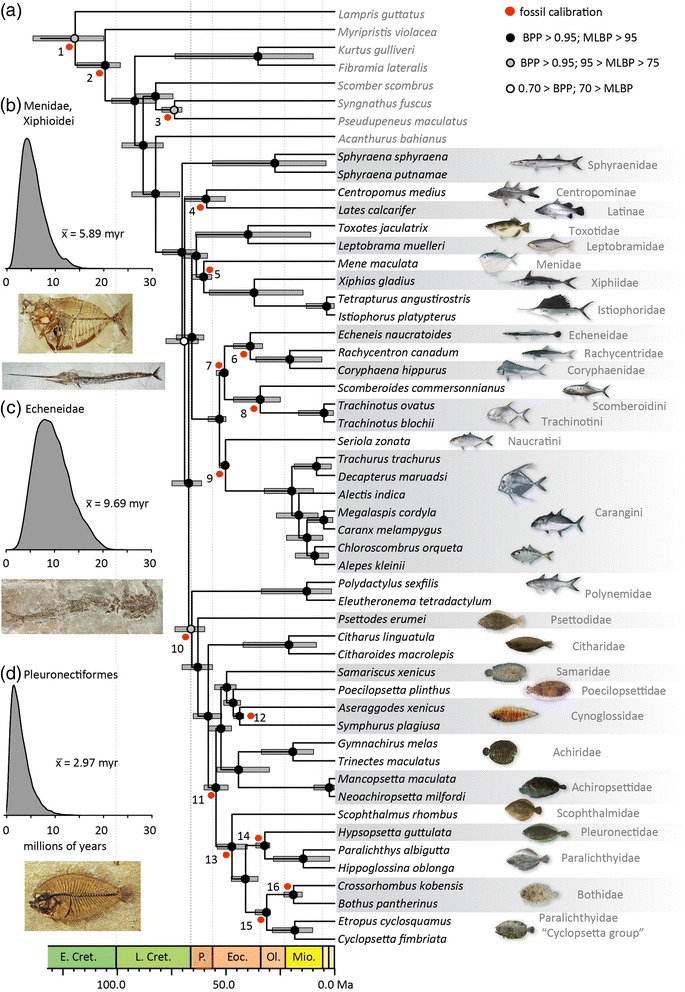
Interrelationships of, and evolutionary timescale for, Carangimorpha based on UCEs. **a** Timescaled tree, based on the topology from the 95 % complete data matrix (596 loci). Discs indicate nodal support as assessed by posterior probability (BPP) and boostrapping (MLBP). Numbered red discs indicate position of fossil calibrations, details of which are given in Additional files 1 and 2. Posterior density plots for the maximum interval over which the bodyplans of **b** billfishes (Xiphioidei) and moonfishes (Menidae); **c** remoras (Echeneidae); and **d** flatfishes (Pleuronectiformes) could have arisen. Images of modern fishes from J.E. Randall, used with permission. Fossil images from M. Friedman

Our sequencing generated an average of 996 loci per taxon. After alignment and alignment trimming, mean locus length was 365 nucleotides (range: 121–811), with a mean of 97 parsimony informative sites per locus. The phylogenetic trees inferred in this study from the concatenated datasets are resolved with strong node support and exhibit near-identical topologies regardless of the matrix examined [75 % (1014 loci), 95 % (596 loci) and 100 % (97 loci) complete] and method of analysis (Bayesian and maximum likelihood) (Fig. 2, Additional file 1: Figure S1). Phylogenies inferred from coalescent gene tree species tree (GT-ST) analyses share a majority of nodes in common with results from concatenated analyses (Additional file 1: Figure S2). Disagreement is localized to the deepest divergences within Carangimorpha, which are poorly supported in GT-ST phylogenies. Best practice for the application of coalescent GT-ST methods to genomic data is currently debated [68, 69], and we therefore base our discussion on the phylogenies inferred using the 95 % complete concatenated matrix (Fig. 2). The population tree generated by BCA matched the topology of the concatenated 95 % complete matrix (Additional file 1: Figure S3). We present the phylogenies inferred using concatenation analysis of the 75 % and 100 % complete data matrices, GT-ST, and BUCKy-generated primary concordance and population trees in the extended data (Additional file 1: Figures S1-S3), and we note disagreements between phylogenies resulting from concatenated and GT-ST analyses.

There is strong support for nearly all nodes in phylogenies inferred from concatenated analyses, with Bayesian posterior probabilities (PP) of >0.99 (approximated as ‘1’ below) and bootstrap support percentages (BP) of 100 %. With the exception of the placement of Sphyraenidae, we find strong support for many of the deepest nodes within Carangimorpha, in contrast to previous studies that have inferred inconsistent and poorly supported relationships among these deeply divergent lineages [3, 4, 15, 38].

### The monophyly and relationships of flatfishes

**Fig. 3:**
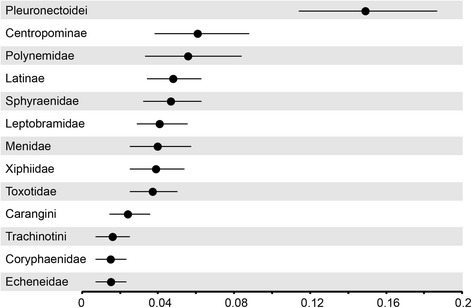
Concordance factors and 95 % CI showing global estimates for the proportion of loci supporting alternative relationships between *Psettodes* and other major carangimorph lineages

Flatfish monophyly is unambiguously supported in phylogenies resulting from all of our concatenated analyses (PP = 1, BP = 100 %), but it also receives strong support in GT-ST analyses, and flatfishes appear as a clade in both the primary concordance and population trees generated by BCA. Concordance factors calculated for the loci containing all fifteen of the subsampled taxa (557 loci contained sequence data for *Psettodes* and the selected representatives of pleuronectoids and each of the remaining major carangimorph lineages) show that a plurality of loci produced gene trees in which *Psettodes* forms a clade with the other two sampled flatfishes, to the exclusion of all other carangimorphs (Fig. 3). The genome-wide BUCKy concordance factor estimate of 0.149 [95%CI: 0.114–0.187] for the *Psettodes* + Pleuronectoidei clade is higher than, and non-overlapping with, concordance factors estimated for any alternative topology in which *Psettodes* is recovered in a clade with any non-flatfish lineage (Fig. 3). Consistent with previous morphological hypotheses [23, 26–28, 70, 71], we resolve *Psettodes* as the sister lineage to Pleuronectoidei, which contains all other flatfish species. The UCE-inferred phylogenies place Citharidae as the sister lineage to all remaining pleuronectoids, in agreement with previous morphological [28, 70] and molecular analyses [4]. Interrelationships among other pleuronectoid families are identical to those reported in a recent molecular phylogenetic analysis of flatfishes [15], with universally high support (PP = 1, BP > 95 %).

### Carangimorph relationships

We resolve Polynemidae as the sister lineage of a monophyletic flatfish clade (Fig. 2). Although this relationship is strongly supported for some datasets (PP ≥ 0.99 across all datasets, BP = 54, 84, and 100 % for 97, 596, and 1014 locus datasets, respectively), it has not been found in any previous molecular study of carangimorphs or on the basis of morphological features (Fig. 1). The clade containing polynemids and flatfishes is the sister lineage of a well-supported (PP = 1, BP = 100 %) and diverse clade containing species from at least nine families: Toxotidae, Leptobramidae (beach salmon), Menidae, Xiphiidae (swordfish), Istiophoridae (marlins and sailfishes), Carangidae (jacks), Rachycentridae (cobia), Coryphaenidae (dolphinfishes), and Echeneidae. Consistent with results from previous morphological [27, 72, 73] and molecular [3, 4, 15] phylogenetic analyses, we find strong support (PP = 1, BP = 100 %) for a carangiform [27] clade including jacks and echeneoids (cobia, dolphinfishes, and remoras). As presented in previous molecular phylogenies, echeneoids are nested within jacks [1–4, 34] rather than the sister lineage of carangids as hypothesized based on anatomical data [73]; we find that the carangid clades Trachinotini and Scomberoidini are more closely related to echeneoids than they are to other jacks [4, 34]. Within echeneoids, we find unambiguous support (PP = 1, BP = 100 %) for a sister-group relationship between cobia and dolphinfishes to the exclusion of remoras [1, 3, 4, 15, 17, 38, 73, 74] rather than the previously hypothesized relationship between more phenotypically similar remoras and cobia [18].

The UCE-inferred phylogeny resolves a clade of anatomically disparate fishes as the sister lineage of carangiforms (Fig. 2). Within this clade, we recover the moonfish *Mene maculata*, classified in the monotypic Menidae, as the sister lineage of xiphioids. This morphologically unanticipated relationship is weakly supported in some previous molecular analyses [4, 34], but receives unambiguous support in the UCE trees (PP = 1, BP = 100 %). Consistent with a clear morphological [7, 75] and molecular [3, 4, 10, 15, 34, 38] consensus, the monophyly of xiphoids, containing Xiphiidae and Istiophoridae, is strongly supported (PP = 1, BP = 100 %). We resolve this new clade containing moonfish and billfishes as the sister lineage of a group uniting *Toxotes* and *Leptobrama*, each of which is the only generic representative of its family (Toxotidae, Leptobramidae). This clade is present in the results of multiple molecular analyses [4, 15, 34], although with weak support.

The only disagreement among the phylogenies from our different concatenated analyses involve the deepest nodes in Carangimorpha, and it specifically concerns the relationships between Sphyraenidae (barracudas), Centropomidae (snooks and Nile perches; comprising Centropominae and Latinae), and all remaining carangimorphs (Fig. 2, Additional file 1: Figure S1). The monophyly of both Centropominae and Latinae is consistently supported in all analyses (PP = 1, BP = 64 % in 97 loci matrix, PP = 1, BP = 100 % in 596 and 1014 matrix analyses), corroborating results from previous morphological [76, 77] and molecular analyses [4, 15, 34, 38, 78]. The phylogenies resulting from analysis of the 97 locus dataset resolve Centropomidae and Sphyraenidae as a clade, which is the sister lineage of all other carangimorphs (PP = 1, BP = 84 %), while the phylogenies inferred using the 596 and 1014 locus datasets place sphyraenids (PP = 0.61, BP = 40 %) and centropomids (PP = 0.57, BP = 44 %), respectively, as successive outgroups to all remaining carangimorphs. Neither of these alternative arrangements is well supported, indicating a degree of phylogenetic uncertainty regarding the earliest divergences within Carangimorpha even in the face of extensive genetic sampling.

The GT-ST results are congruent with nearly all major aspects of the phylogenies inferred from concatenated data (Additional file 1: Figures S1 and S2). The placement of polynemids represents the most conspicuous difference between phylogenies inferred using concatenated and GT-ST methods. The ASTRAL-inferred GT-ST resolves threadfins as the sister lineage of the clade including toxotids, leptobramids, menids, xiphioids, and carangiforms, rather than pleuronectiforms as in concatenated analyses, while BUCKy-inferred population tree places Polynemidae as the sister of flatfishes. We note here, however, that the concordance factors for conflicting placement of Polynemidae have overlapping 95 % credible intervals. Although GT-ST analyses provide strong support for the monophyly of Carangimorpha and Pleuronectiformes, deep divergences among major carangimorph lineages are not well supported.

### A timescale for the diversification of Carangimorpha

Our multiple relaxed-clock molecular dating analyses produced similar and consistent estimates of divergence dates across different subsamples of UCE loci and between both analytical frameworks in the BEAST and PAML software packages, with overlapping 95 % highest posterior density ranges for all nodes (Fig. 2, Additional file 1: Figure S4, Additional file 1: Table S3). We refer to specific branch length and divergence date estimates derived from analysis of one set of 75 UCE loci (depicted in Fig. 2); but our BEAST analyses of three other 75 UCE-loci-datasets and PAML analysis of 596 loci provide very similar node ages, branch lengths, and overlapping 95 % highest posterior densities. We infer an origin of Carangimorpha at 71.0 Ma, shortly before the end of the Late Cretaceous, although we cannot reject an earliest Paleocene (Danian) origin [95 % highest posterior density (HPD) interval: 63.44, 80.86]. Most of the strikingly anatomically divergent carangimorph lineages arose in the subsequent 15 million years, before the end of the Paleocene. This is consistent with previous molecular phylogenetic studies [3, 4] (but see Santini and Carnevale [79]) as well as the earliest fossil occurrences of specialized lineages like billfishes, jacks, flatfishes, menids, and barracudas by the end of the first stage of the Eocene [29, 80].

We estimate the pleuronectiform crown node at 61.3 Ma [95 % HPD: 54.3–69.5 Ma]. The most recent common ancestor of flatfishes and threadfins, their living symmetrical sister lineage, is 65.7 Ma [95 % HPD: 57.3–72.6 Ma]. The mean length of the flatfish stem across our posterior sample of trees is 2.97 Myr [median: 2.45 Myr; 95 % HPD: 0.47–8.35 Myr], and it provides the longest possible span over which flatfish asymmetry could have evolved. Maximum timelines for the origin of other remarkable carangimorph bodyplans are generally longer. The divergence between Menidae and Xiphioidei is estimated as 60.6 Ma [median: 95 % HPD: 56.5–66.6 Ma]. Early fossil representatives of both lineages constrain the origin of their unique bodyplans to no later than the earliest Eocene [80, 81]. Thus the mean maximum time for origin of both bodyplans is 5.89 Myr [median: 5.42 Myr; 95 % HPD: 1.91–12.48 Myr]. The difference in age between crown Echeneoidei (marking the divergence of remoras from other echeneoids) and the oldest anatomically modern remora fossils give a maximum time over which the remora adhesion disc evolved. Fossils with discs closely resembling those of modern species are known from the early Oligocene [17, 82, 83], giving a maximum evolutionary interval of 9.69 Myr [median: 9.27 Myr; 95 % HPD: 3.87–17.68 Myr]. Our ability to constrain the time over which other distinctive carangimorph bodyplans—like those of polynemids and toxotids—arose is limited by sparse fossil records [80] and sampling of modern lineages that is insufficient to constrain crown ages.

## Discussion

### The flatfish monophyly challenge

The recent series of studies that report conflicting interpretations of carangimorph phylogenies inferred from multi-locus sequence data and their apparent equivocal support for the monophyly (or polyphyly) of flatfishes have breathed new life into old debates about the evolutionary origins of their asymmetrical bodyplan [31–33, 84]. Our results generated from more than 1000 UCE loci provide strong molecular support for the monophyly of flatfishes and the single origin of cranial asymmetry, regardless of analytical framework applied (Fig. 2, Additional file 1: Figures S1-S3). Analysis of concordance among loci provides further insight into the longstanding difficulty of resolving relationships among carangimorph lineages using smaller molecular datasets that may not be apparent from nodal support values generated by concatenation or species tree analyses. Concordance factors calculated from our dataset estimate that a genome-wide proportion of 14.9 % of loci produce gene trees that reflect a single flatfish clade. In contrast, the estimated frequency for alternative topologies in which *Psettodes* is recovered in clades with non-flatfishes is significantly lower and non-overlapping with a monophyletic flatfish scenario (Fig. 3). Prior to this study, the largest dataset of multiple unlinked loci applied to carangimorphs [34] found weak support from concatenated analysis of 23 loci for a flatfish clade (PP = 0.65), and attributed the low nodal support to nucleotide compositional bias in protein coding genes and to a lesser extent, ILS. The proportion of loci examined by [34] that recover a flatfish clade (3 out of 23 loci, or 13 %) is within the 95 % credible interval estimated from our analyses of UCE loci (95 % CI of 11.4–18.7 %). While other phenomena (such as the non-stationarity nucleotide composition as identified by [34]) may introduce error into gene tree estimation, the short internal branches subtending successive divergences of carangimorph lineages likely resulted in substantial ILS, making their relationships difficult to recover with small datasets.

**Fig. 4:**
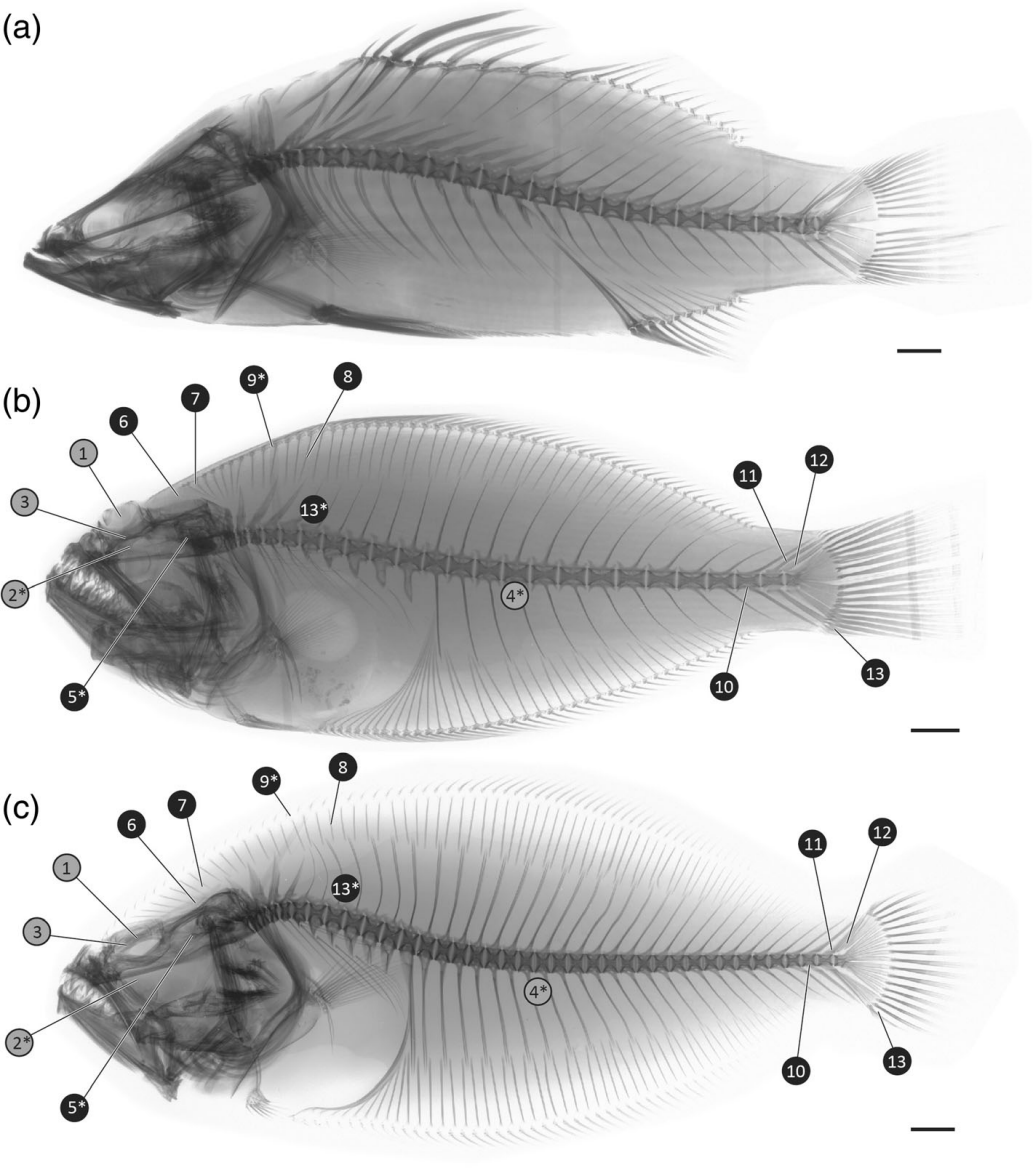
Morphological evidence for flatfish monophyly is not restricted to cranial asymmetry, but is instead widely distributed in both hard-tissue and soft-tissue anatomy. **a** inverted radiograph of *Lates calcarifer* (Natural History Museum, London [NHMUK] 5.85), a carangimorph retaining many generalized percomorph features. **b** inverted radiograph of *Psettodes erumei* (NHMUK 1931.4.23.2), a member of Psettodoidei. **c** inverted radiograph of *Paralichthys albigutta* (NHMUK 1989.9.22.78-81), a member of Pleuronectoidei. Proposed pleuronectiform synapomorphies related to cranial asymmetry indicated in grey: 1, orbital migration; 2*, *recessus orbitalis*; 3, pseudomesial bar; 4*, asymmetrical pigmentation. Proposed pleuronectiform synapomorphies not related to cranial asummetry indicated in black: 5*, circumsulcal depression on inner face of saccular otolith; 6, dorsal-fin insertion above skull; 7, absence of supraneurals; 8, absence of membranous extensions on shafts of most dorsal- and anal-fin proximal-middle radials; 9*, epaxial muscle insertions on dorsal-fin proximal-middle radials comprising bundles of muscle that pass underneath the depressors dorsales; 10, haemal arch and spine of third preural vertebra fused to the centrum; 11, full neural spine on the second preural centrum; 12, two or fewer epurals; 13, absence of procurrent spur; 14*, partial or complete fusion between the dorsal fin longitudinal ramus and the dorsal longitudinal collector nerve of the trunk lateral line nervous system. Characters marked with an asterisk (‘*’) indicate soft-tissue features not apparent in radiographs. See references [26, 28, 71, 86, 87] for a discussion of proposed synapomorphies. Scalebars represent 10 mm

The strong support for flatfish monophyly obtained from our molecular analyses of UCE loci bolsters the morphological consensus that this remarkable innovation evolved only once. The anatomical argument for flatfish monophyly has been caricatured as reliant almost exclusively on cranial asymmetry, and thus a hypothesis formed from limited evidence. However, probable pleuronectiform synapomorphies have been identified across multiple anatomical systems, including the axial skeleton [28, 71], caudal-fin endoskeleton [28, 71], otoliths [85], patterns of epaxial muscle insertions [86], and innervations of the trunk lateral line [87]; (Fig. 4). These synapomorphies collectively represent a strong anatomical case for flatfish monophyly, independent of cranial asymmetry.

### Morphological support for relationships within carangimorpha

Although our phylogeny agrees with many aspects of previous morphological classifications (e.g., the monophyly of xiphioids [7, 75], centropomids [77], pleuronectiforms [26], and echeneoids [73]), it also reveals many unanticipated but strongly supported sister-group relationships, some of which have appeared in previous molecular phylogenies. There have been limited efforts to discover morphological evidence uniting anatomically disparate carangimorph lineages, and existing studies have been hampered by ambiguities in character polarity [71] and limited taxonomic comparisons [10].

Patterns of lineage diversification within carangimorphs suggest that unambiguous morphological support for some clades may prove elusive. Our molecular clock analyses indicate many specialized carangimorph groups have independent evolutionary histories that are considerably longer than those shared uniquely with their immediate sister taxa [3, 4, 38]. As a result, there was little time over which traits providing evidence for sister-group relationships could evolve relative to the time that any synapomorphies might be overwritten by the profound morphological specializations characteristic of individual carangimorph lineages. Nevertheless, it is clear that considerable anatomical evidence for monophyly has accumulated along some of the shortest branches within the carangimorph phylogeny. This is particularly apparent for the ca. 3 Myr-long flatfish stem (discussed above).

More careful anatomical scrutiny may yield evidence for the phylogenetic relationships in the UCE-inferred trees (Fig. 2; Additional file 1: Figures S1-S3), especially because many of the sister-group pairings within Carangimorpha have never been seriously considered—and therefore investigated—from a morphological perspective. An anatomically unanticipated sister-group relationship between billfishes and the moonfish *Mene* is a common motif of carangimorph molecular phylogenies, and one for which we recovered strong support in the UCE-inferred trees (e.g., Fig. 2). The anatomical specializations of these groups result in strikingly different “baupläne” that may have discouraged close comparison in the past, although we note a series of derived features common to both lineages (for details of comparative materials, see Additional file 1: Table S2): considerable elongation of the second and third pelvic-fin rays (pelvic fins are lost in xiphiids), caudal hypurostegy, a consolidated hypural plate arising from the fusion of hypurals 1-4 to one another and the ural centrum (further fusion characterizes istiophorids), and posterior extensions of the gas bladder [88–90]. Similarly, the sister-group relationship between *Leptobrama* and toxotids was previously unpredicted on the basis of the morphology, but the two groups share a number of unusual features among carangimorphs: presence of endopterygoid teeth (uniquely), presence of ectopterygoid teeth (with Polynemidae), and presence of more anal-fin rays than dorsal-fin rays (with Polynemidae and Lactariidae [not analysed in current study]) [90, 91]. These observations are joined by sporadic reports of shared specializations in other carangimorph lineages such as similar larval colour patterns in billfishes and sphyraenids [92]; prenasal canal units in polynemids, toxotids, and carangiforms, with ossified prenasals in the latter two [72]; and a series of specializations common to *Mene*, *Lactarius* and many carangiforms [91]. A systematic anatomical survey is required to determine whether the shared morphological similarities noted in this and previous studies corroborate the novel and strongly supported relationships in the carangimorph molecular phylogeny.

### The origin of flatfish asymmetry: gradual but rapid

Our time-calibrated phylogeny provides the first robust constraints for the timescale over which the flatfish transformation occurred. On average, we conclude that complete orbital migration arose in no more than 2.97 Myr, although we cannot reject the possibility that it may have taken less than 470 kyr or as long as 7.96 Myr based on our posterior sample of trees. All stem pleuronectiforms identified to date show incomplete orbital migration [28, 71]. However, the identification of extinct flatfishes showing complete asymmetry but which branch outside the crown would further reduce the length of the stem over which modern pleuronectiform cranial geometry arose, thereby increasing the rate of this evolutionary transformation. Comparisons both within and outside Carangimorpha provide context for the rapid origin of the flatfish bodyplan. Mean maximum timescales for the evolution of billfishes, moonfishes, and remoras are on the order of 5–10 Myr, two to three times longer than comparable estimates for the origin of flatfish asymmetry. Similarly, the timescale of a few million years for the origin of flatfish asymmetry compares closely with the estimated age of some cichlid radiations in African rift lakes [93], upheld as examples of explosive evolutionary diversification [94], although flatfishes are arguably a more extreme morphological departure from standard acanthomorph bodyplans than even the most peculiar modern cichlids [95]. Outside of fishes, the timescale for the evolution of pleuronectiform asymmetry is substantially shorter than those estimated for the origins of the bodyplans associated with whales (ca. 20 Myr [96]) and anatomically modern humans (ca. 7 Myr). We hypothesize that the rapid evolution of flatfish asymmetry might reflect a steep peak associated with a new adaptive zone, as classically hypothesized for other rapid divergences by Simpson [97].

### Diversification of carangimorpha

Our well-supported phylogeny provides new insight for exploring the origin of the anatomically diverse lineages that comprise Carangimorpha. The earliest diverging carangimorph lineage includes what are arguably the most anatomically ‘generalized’ members of the radiation: the superficially perch-like snooks and Nile perches. Interestingly, the two lineages constituting the principal carangimorph clade, excluding centropomids and sphyraenids, are differentiated along ecological lines. The first unnamed lineage is broadly associated with benthic environments, and its two constituent clades show striking adaptations to life at or near the sediment-water interface: profound asymmetry in flatfishes and free pectoral-fin rays in polynemids that serve a tactile function [19]. By contrast, the second major lineage contains pelagic groups like jacks, billfishes, and dolphinfishes. Our discovery of broad environmental divisions within carangimorphs represents the latest example of molecular analyses revealing novel clades of percomorph teleosts that share a common habitat preference or ecology [74]. The major ecological split in carangimorph phylogeny mirrors patterns apparent at smaller spatial and temporal scales in fishes, in which initial divergences within populations often reflect partitioning between benthic and pelagic habitats and resources [98].

Our analyses suggest that anatomically modern bodyplans evolved by the early Eocene (ca. 50 Ma), following major divergences in the latest Late Cretaceous and Paleocene. The rich fossil records of many individual groups [29], combined with our well-supported time-calibrated genomic perspective on phylogenetic relationships, make carangimorphs an ideal system for studying patterns of phenotypic diversification over spatial and temporal scales not reflected by young, geographically restricted clades that are often the focus of research on adaptive radiation [94, 99, 100]. The study of ancient marine percomorph radiations like the ecologically varied carangimorphs, pelagic scombriforms [74], and Antarctic nototheneoids [101] may provide insights into the generation of biological diversity that is persistent over long evolutionary timescales not provided by model systems like sticklebacks and cichlids that have diversified in spatially limited and geologically ephemeral environments [99, 102].

## Conclusion

The invariably low support for monophyly of flatfishes found in previous molecular phylogenetic analyses is emblematic of a common problem in reconstructing the evolutionary history of rapidly diverging lineages throughout the Tree of Life. Although increasingly larger gene-by-gene datasets have provided valuable discoveries regarding the relationships among acanthomorph fishes and the timing of their divergences [3, 4], these datasets may not be large enough to overcome discordance due to phenomena such as incomplete lineage sorting for nodes within rapidly branching portions of the acanthomorph phylogeny. The resolution of carangimorph relationships provided by high throughput sequencing of UCE loci serves as an improved framework on which to study the evolution and diversification of fish bodyplans, and our results suggest similar phylogenomic approaches will be necessary to resolve historically difficult nodes in the acanthomorph phylogeny, as in the case of the flatfishes.

## Additional files

Additional file 1: Extended descriptions of protocols for DNA isolation, library preparation, sequencing, and data pipelining, and Additional file 1: Figures S1-S4. (DOCX 6704 kb)
Additional file 2: Descriptions of fossil calibrations used in divergence dating analyses, including details of age priors and the fossil data upon which they are based. (DOCX 92 kb)

## References

[1] Smith WL, Wheeler WC (2006). Venom evolution widespread in fishes: A phylogenetic road map for the bioprospecting of piscine venoms. J Hered.

[2] Li B, Dettai A, Cruaud C, Couloux A, Desoutter-Meniger M, Lecointre G (2009). RNF213, a new nuclear marker for acanthomorph phylogeny. Mol Phylogen Evol.

[3] Near TJ, Dornburg A, Eytan RI, Keck BP, Smith WL, Kuhn KL, Moore JA, Price SA, Burbrink FT, Friedman M (2013). Phylogeny and tempo of diversification in the superradiation of spiny-rayed fishes. Proc Natl Acad Sci U S A.

[4] Betancur-R R, Broughton RE, Wiley EO, Carpenter K, Lopez JA, Li C, Holcroft NI, Arcila D, Sanciangco M, Cureton Ii JC, et al. The tree of life and a new classification of bony fishes. PLoS Curr. 2013; doi:10.1371/currents.tol.53ba26640df0ccaee75bb165c8c26288. 10.1371/currents.tol.53ba26640df0ccaee75bb165c8c26288 PMC3644299 23653398

[5] Faircloth BC, Sorenson L, Santini F, Alfaro ME (2013). A phylogenomic perspective on the radiation of ray-finned fishes based upon targeted sequencing of Ultraconserved Elements (UCEs). PLoS One.

[6] Phylogenetic Classification of Bony Fishes - Version 3 (http://www.deepfin.org/Classification_v3.htm) Accessed 21 Jan 2016.

[7] Collette BB, Potthoff T, Richards WJ, Ueyanagi S, Russo JL, Nishikawa Y, Moser HG, Richards WJ, Cohen DM, Fahay MP, Kendall AW, Richardson SL (1984). Scombroidei: development and relationships. Ontogeny and Systematics of Fishes.

[8] Kaufman L, Liem KF (1982). Fishes of the suborder Labroidei (Pisces: Perciformes): phylogeny, ecology, and evolutionary significance. Breviora.

[9] Orrell TM, Collette BB, Johnson GD (2006). Molecular data support separate scombroid and xiphioid clades. Bull Mar Sci.

[10] Little AG, Lougheed SC, Moyes CD (2010). Evolutionary affinity of billfishes (Xiphiidae and Istiophoridae) and flatfishes (Plueronectiformes): Independent and trans-subordinal origins of endothermy in teleost fishes. Mol Phylogen Evol.

[11] Wainwright PC, Smith WL, Price SA, Tang KL, Sparks JS, Ferry LA, Kuhn KL, Eytan RI, Near TJ (2012). The evolution of pharyngognathy: a phylogenetic and functional appraisal of the pharyngeal jaw key innovation in labroid fishes and beyond. Syst Biol.

[12] Smith WL, Craig MT (2007). Casting the percomorph net widely: the importance of broad taxonomic sampling in the search for the placement of serranid and percid fishes. Copeia.

[13] Miya M, Takeshima H, Endo H, Ishiguro NB, Inoue JG, Mukai T, Satoh TP, Yamaguchi M, Kawaguchi A, Mabuchi K (2003). Major perspectives of higher teleostean phylogenies: a new perspective based on 100 complete mitochondrial DNA sequences. Mol Phylogen Evol.

[14] Chen WJ, Bonillo C, Lecointre G (2003). Repeatability of clades as a criterion of reliability: a case study for molecular phylogeny of Acanthomorpha (Teleostei) with larger number of taxa. Mol Phylogen Evol.

[15] Betancur-R R, Li C, Munroe TA, Ballesteros JA, Ortí G (2013). Addressing gene tree discordance and non-stationarity to resolve a multi-locus phylogeny of the flatfishes (Teleostei: Pleuronectiformes). Syst Biol.

[16] Block BA, Finnerty JR (1994). Endothermy in fishes: a phylogenetic analysis of constraints, predispositions, and selection pressures. Environ Biol Fishes.

[17] Friedman M, Johanson Z, Harrington RC, Near TJ, Graham MR (2013). An early fossil remora (Echeneoidea) reveals the evolutionary assembly of the adhesion disc. Proc R Soc Lond [Biol].

[18] O’Toole B (2002). Phylogeny of the species of the superfamily Echeneoidea (Perciformes : Carangoidei: Echeneidae, Rachycentridae, and Coryphaenidae), with an interpretation of echeneid hitchhiking behaviour. Can J Zool-Rev Can Zool.

[19] Motomura H, Sado T, Kimura S (2002). Feeding behaviour of *Polydactylus plebeius* (Perciformes: Polynemidae) in an aquarium. Jap J Ichthyol.

[20] Halstead BW, Danielson DD, Baldwin WJ, Engen PC (1972). Morphology of the venom apparatus of the leatherback fish *Scomberoides sanctipetri*(Cuvier). Toxicon.

[21] Timmermans PJ (2000). Prey catching in the archer fish: marksmanship, and endurance of squirting at an aerial target. Neth J Zool.

[22] Norman JR (1934). A systematic monograph of the flatfishes (Heterostomata), Vol. 1 Psettodidae, Bothidae, Pleuronectidae.

[23] Hubbs CL (1945). Phylogenetic position of the Citharidae, a family of flatfishes. Miscellaneous Publications Museum of Zoology, University of Michigan.

[24] Lauder GV, Liem KF (1983). The evolution and interrelationships of the actinopterygian fishes. Bull Mus Comp Zool.

[25] Hensley DA, Ahlstrom EH, Moser H, Richards WJ, Cohen DM, Fahay MP, Kendall AW, Richardson SL (1984). Pleuronectiformes: relationships. Ontogeny and Sytematics of Fishes.

[26] Chapleau F (1993). Pleuronectiform relationships: A cladistic reassessment. Bull Mar Sci.

[27] Wiley EO, Johnson GD, Nelson JS, Schultze H-P, Wilson MVH (2010). A teleost classification based on monophyletic groups. Origin and phylogenetic interrelationships of teleosts.

[28] Friedman M (2008). The evolutionary origin of flatfish asymmetry. Nature.

[29] Friedman M (2010). Explosive morphological diversification of spiny-finned teleost fishes in the aftermath of the end-Cretaceous extinction. Proc R Soc Lond [Biol].

[30] Regan CT (1910). The origin and evolution of the teleostean fishes of the order Heterosomata. Ann Mag Nat Hist.

[31] Kyle HM (1921). The asymmetry, metamorphosis and origin of flat-fishes. Philos Trans R Soc London [Biol].

[32] Chabanaud P (1949). Le problem de la phylogenése des Heterostomata. Bull Inst Océanogr Monaco.

[33] Amaoka K (1969). Studies on the sinistral flounders found in the waters around Japan. Taxonomy, anatomy and phylogeny. J Shimonoseki Univ Fish.

[34] Betancur-R R, Ortí G (2014). Molecular evidence for the monophyly of flatfishes (Carangimorpharia: Pleuronectiformes). Mol Phylogen Evol..

[35] Campbell M, Lopez JA, Satoh TP, Chen WJ, Miya M (2014). Mitochondrial genomic investigation of flatfish monophyly. Gene.

[36] Sanciangco MD, Carpenter KE, Betancur-R R (2016). Phylogenetic placement of enigmatic percomorph families (Teleostei: Percomorphaceae). Mol Phylogen Evol.

[37] Chanet B (1997). A cladistic reappraisal of the fossil flatfishes record consequences on the phylogeny of the Pleuronectiformes (Osteichthyes: Teleostei). Ann Sci Nat.

[38] Campbell MA, Chen WJ, Lopez JA (2013). Are flatfishes (Pleuronectiformes) monophyletic?. Mol Phylogen Evol.

[39] Campbell MA, Chen WJ, Lopez JA (2014). Molecular data do not provide unambiguous support for the monophyly of flatfishes (Pleuronectiformes): a reply to Betancur-R and Ortí. Mol Phylogen Evol..

[40] Foster PG, Hickey DA (1999). Compositional bias may affect both DNA-based and protein-based phylogenetic reconstructions. J Mol Evol.

[41] Felsenstein J (1978). Cases in Which Parsimony or Compatibility Methods Will Be Positively Misleading. Syst Zool.

[42] Anderson FE, Swofford DL (2004). Should we be worried about long-branch attraction in real data sets? Investigations using metazoan 18S rDNA. Mol Phylogen Evol.

[43] Maddison WP (1997). Gene trees in species trees. Syst Biol.

[44] Edwards SV, Liu L, Pearl DK (2007). High-resolution species trees without concatenation. Proc Natl Acad Sci U S A.

[45] Kubatko LS (2009). Identifying hybridization events in the presence of coalescence via Model selection. Syst Biol.

[46] Degnan JH, Rosenberg NA (2006). Discordance of species trees with their most likely gene trees. PLoS Genet.

[47] Maddison WP, Knowles LL (2006). Inferring phylogeny despite incomplete lineage sorting. Syst Biol.

[48] McGee MD, Faircloth BC, Borstein SR, Zheng J, Hulsey CD, Wainwright PC, Alfaro ME. Replicated divergence in cichlid radiations mirrors a major vertebrate innovation. P Roy Soc B-Biol Sci. 2016;283(1822). 10.1098/rspb.2015.1413 PMC4721080 26763694

[49] Gilbert PS, Chang J, Pan C, Sobel EM, Sinsheimer JS, Faircloth BC, Alfaro ME (2015). Genome-wide ultraconserved elements exhibit higher phylogenetic informativeness than traditional gene markers in percomorph fishes. Mol Phylogenet Evol.

[50] Hosner PA, Faircloth BC, Glenn TC, Braun EL, Kimball RT (2016). Avoiding missing data biases in phylogenomic inference: an empirical study in the landfowl (Aves: Galliformes). Mol Biol Evol.

[51] Streicher JW (2016). Schulte 2nd JA, Wiens JJ. How should genes and taxa be sampled for phylogenomic analyses with missing data? An empirical study in iguanian lizards. Syst Biol.

[52] Stamatakis A (2006). RAxML-VI-HPC: Maximum likelihood-based phylogenetic analyses with thousands of taxa and mixed models. Bioinformatics.

[53] Lanfear R, Calcott B, Ho SY, Guindon S (2012). Partitionfinder: combined selection of partitioning schemes and substitution models for phylogenetic analyses. Mol Biol Evol.

[54] Lanfear R, Calcott B, Kainer D, Mayer C, Stamatakis A. Selecting optimal partitioning schemes for phylogenomic datasets. BMC Evol Biol. 2014;14. 10.1186/1471-2148-14-82 PMC4012149 24742000

[55] Aberer AJ, Kobert K, Stamatakis A (2014). ExaBayes: massively parallel Bayesian tree inference for the whole-genome era. Mol Biol Evol.

[56] Rambaut A, Suchard MA, Xie D, Drummond AJ: Tracer v1.6. In.: Available from http://beast.bio.ed.ac.uk/Tracer; 2014.

[57] Mirarab S, Reaz R, Bayzid MS, Zimmermann T, Swenson MS, Warnow T (2014). ASTRAL: genome-scale coalescent-based species tree estimation. Bioinformatics.

[58] Kubatko LS, Degnan JH (2007). Inconsistency of phylogenetic estimates from concatenated data under coalescence. Syst Biol.

[59] Ane C, Larget B, Baum DA, Smith SD, Rokas A (2007). Bayesian estimation of concordance among gene trees. Mol Biol Evol.

[60] Larget BR, Kotha SK, Dewey CN, Ane C (2010). BUCKy: gene tree/species tree reconciliation with Bayesian concordance analysis. Bioinformatics.

[61] Degnan JH, DeGiorgio M, Bryant D, Rosenberg NA (2009). Properties of consensus methods for inferring species trees from gene trees. Syst Biol.

[62] Ronquist F, Teslenko M, van der Mark P, Ayres DL, Darling A, Hohna S, Larget B, Liu L, Suchard MA, Huelsenbeck JP (2012). MrBayes 3.2: efficient Bayesian phylogenetic inference and model choice across a large model space. Syst Biol.

[63] Drummond AJ, Suchard MA, Xie D, Rambaut A (2012). Bayesian phylogenetics with BEAUti and the BEAST 1.7.. Mol Biol Evol.

[64] Yang ZH (2007). PAML 4: Phylogenetic analysis by maximum likelihood. Mol Biol Evol.

[65] Hedman MM (2010). Constraints on clade ages from fossil outgroups. Paleobiology.

[66] Paradis E, Claude J, Strimmer K (2004). APE: Analyses of phylogenetics and evolution in R language. Bioinformatics.

[67] Sukumaran J, Holder MT (2010). DendroPy: a Python library for phylogenetic computing. Bioinformatics.

[68] Gatesy J, Springer MS (2014). Phylogenetic analysis at deep timescales: Unreliable gene trees, bypassed hidden support, and the coalescence/concatalescence conundrum. Mol Phylogen Evol.

[69] Edwards SV, Xi ZX, Janke A, Faircloth BC, McCormack JE, Glenn TC, Zhong BJ, Wu SY, Lemmon EM, Lemmon AR (2016). Implementing and testing the multispecies coalescent model: A valuable paradigm for phylogenomics. Mol Phylogen Evol.

[70] Hoshino K (2001). Monophyly of the Citharidae (Pleuronectoidei: Pleuronectiformes: Teleostei) with considerations of pleuronectid phylogeny. Ichthyol Res.

[71] Friedman M (2012). Osteology of †*Heteronectes chaneti* (Acanthomorpha, Pleuronectiformes), an Eocene stem flatfish, with a discussion of flatfish sister-group relationships. J Vert Paleontol.

[72] Freihofer WC (1978). Cranial nerves of a percoid fish, *Polycentrus schomburgkii* (Family Nandidae), a contribution to the morphology and classification of the order Perciformes. Occas Pap, Calif Acad Sci.

[73] Johnson GD, Moser HG, Richards WJ, Cohen DM, Fahay MP, Kendall AW, Richardson SL (1984). Percoidei: development and relationships. Ontogeny and Systematics of Fishes.

[74] Miya M, Friedman M, Satoh TP, Takeshima H, Sado T, Iwasaki W, Yamanoue Y, Nakatani M, Mabuchi K, Inoue JG (2013). Evolutionary origin of the Scombridae (tunas and mackerels): members of a paleogene adaptive radiation with 14 other pelagic fish families. PLoS One.

[75] Johnson GD (1986). Scombroid phylogeny: an alternative hypothesis. Bull Mar Sci.

[76] Fraser TH (1968). Comparative osteology of the Atlantic snooks (Pisces, *Centropomus*). Copeia.

[77] Greenwood PH (1976). A review of the family Centropomidae (Pisces, Perciformes). Bull Br Mus Nat Hist (Zool).

[78] Li C, Betancur-R R, Smith WL, Ortí G (2011). Monophyly and interrelationships of Snook and Barramundi (Centropomidae sensu Greenwood) and five new markers for fish phylogenetics. Mol Phylogen Evol.

[79] Santini F, Carnevale G (2015). First multilocus and densely sampled timetree of trevallies, pompanos and allies (Carangoidei, Percomorpha) suggests a Cretaceous origin and Eocene radiation of a major clade of piscivores. Mol Phylogen Evol.

[80] Patterson C, Benton MJ (1993). Osteichthyes: Teleostei. The Fossil Record 2.

[81] Friedman M, Johnson GD (2005). A new species of *Mene* (Perciformes: Menidae) from the Paleocene of South America, with notes on the paleoenvironment and a brief review of menid fishes. J Vert Paleontol.

[82] Friedman M, Johanson Z, Harrington RC, Near TJ, Graham MR (2014). On fossils, phylogenies and sequences of evolutionary change. Proc R Soc Lond [Biol].

[83] Micklich N, Gregorová R, Bannikov AF, Baciu DS, Grădianu I, Carnevale G. *Oligoremora rhenana* n. g. n. sp., a new echeneid fish (Percomorpha, Echeneoidei) from the Oligocene of the Grube Unterfeld (“Frauenweiler”) clay pit. PalZ. 2016;90:561-92.

[84] Darwin C (1872). On the Origin of Species by Means of Natural Selection.

[85] Schwarzhans W (1993). A comparative morphological treatise of recent and fossil otoliths of the family Sciaenidae (Perciformes).

[86] Mooi RD, Gill AC (1995). Association of epaxial musculature with dorsal-fin pterygiophores in acanthomorph fishes, and its phylogenetic significance. Bull Br Mus Nat Hist (Zool).

[87] Fukada E, Nakae M, Asoaoka R, Sasaki K (2010). Branching patterns of trunk lateral line nerves in Pleuronectiformes: uniformity and diversity. Ichthyol Res.

[88] Nakamura I (1983). Systematics of the billfishes (Xiphiidae and Istiophoridae). Publ Seto Mar Biol Lab.

[89] Woodland DJ, Carpenter KE, Niem V (2001). Menidae. The Living Marine Resources of the Western Central Pacific.

[90] Tominaga Y (1965). The internal morphology and systematic position of *Leptobrama mülleri*, formerly included in the family Pempheridae. Jap J Ichthyol.

[91] Leis JM (1994). Larvae, adults and relationships of the monotypic perciform fish family Lactariidae. Rec Aust Mus.

[92] Baldwin CC (2013). The phylogenetic significance of colour patterns in marine teleost larvae. Zool J Linn Soc.

[93] Friedman M, Keck BP, Dornburg A, Eytan RI, Martin CH, Hulsey CD, Wainwright PC, Near TJ (2013). Molecular and fossil evidence place the origin of cichlid fishes long after Gondwanan rifting. Proc R Soc Lond [Biol].

[94] Kocher TD (2004). Adaptive evolution and explosive speciation: the cichlid fish model. Nature Rev Genet.

[95] Stewart TA, Albertson RC (2010). Evolution of a unique predatory feeding apparatus: functional anatomy, development and a genetic locus for jaw laterality in Lake Tanganyika scale-eating cichlids. BMC Biol.

[96] Gatesy J, Geisler JH, Chang J, Buell C, Berta A, Meredith RW, Springer MS, McGowen MR (2013). A phylogenetic blueprint for a modern whale. Mol Phylogen Evol.

[97] Simpson GG (1944). Tempo and Mode in Evolution.

[98] Schluter D (1996). Ecological speciation in postglacial fishes. Philos Trans R Soc London [Biol].

[99] Schluter D (1993). Adaptive radiation in sticklebacks - size, shape, and habitat use efficiency. Ecology.

[100] Sato A, Tichy H, O’HUigin C, Grant PR, Grant BR, Klein J (2001). On the origin of Darwin’s finches. Mol Biol Evol.

[101] Near TJ, Dornburg A, Kuhn KL, Eastman JT, Pennington JN, Patarnello T, Zane L, Fernandez DA, Jones CD (2012). Ancient climate change, antifreeze, and the evolutionary diversification of Antarctic fishes. Proc Natl Acad Sci U S A.

[102] Wagner CE, Harmon LJ, Seehausen O (2012). Ecological opportunity and sexual selection together predict adaptive radiation. Nature.

[103] Harrington RC, Faircloth BC, Eytan RI, Smith WL, Near TJ, Alfaro ME, Friedman M (2016). Data from: Phylogenomic analysis of carangimorph fishes reveals flatfish asymmetry arose in a blink of the evolutionary eye.

