# Supplementary material for: Phylogenomic analysis of carangimorph fishes reveals flatfish asymmetry arose in a blink of the evolutionary eye
Source: BMC Evol Biol. 2016 Oct 21;16:224. doi: 10.1186/s12862-016-0786-x (PMC5073739; doi:10.1186/s12862-016-0786-x)
Supplement: Additional file 1: — Extended descriptions of protocols for DNA isolation, library preparation, sequencing, and data pipelining, and Additional file 1: Figures S1-S4. (DOCX 6704 kb) [file 12862_2016_786_MOESM1_ESM.docx]

Supplementary Methods

**1. Library preparation**

We used Qiagen DNeasy Tissue kits following manufacturer’s protocol to isolate DNA from tissue biopsies collected from 55 species (Supplementary Table 1). We did not obtain DNA samples of adequate quality and/or quantity for *Lactarius lactarius*, *Nematistius pectoralis, Pseudopleuronectes americanus, Psettodes belcheri, Solea lascaris,* and *Symphurus diomedianus*. Following DNA extraction, we quantified 2 µL of each template using a Qubit fluorometer (Life Technologies) and we randomly sheared DNA to a target size of 400-600 bp using a sonicator (Diagenode BioRuptor). We input 110-1,100 ng (736 ng mean) into a commercial DNA library preparation kit (Kapa Biosystems) that incorporated “with-bead” ([Fisher, et al. 2011](#_ENREF_7)) cleanup steps during end-repair, +A addition, and ligation steps. We substituted adapters (12 µM) containing a custom index sequence ([Faircloth and Glenn 2012](#_ENREF_6)) for the standard Illumina adapters, and we used a generic SPRI substitute ([Rohland and Reich 2012; hereafter SPRI](#_ENREF_15)) in place of commercially available AMPure reagent (Beckman-Coulter) for all AMPure cleanup steps. Following adapter ligation, we performed two cleanups of the ligation reaction per the manufacturer’s instructions, and we quantified 2 µL of the resulting library using a Qubit fluorometer. We amplified 15 µL of the library volume using a reaction mix of 25 µL HiFi HotStart ReadyMix (Kapa Biosystems), 5 µL of Illumina TruSeq primer mix (5 µM each), and 5 µL ddH20 and the following thermal profile: 98 °C for 45s; 10-12 cycles of 98 °C for 15s, 60 °C for 30s, 72 °C for 60s; and a final extension of 72 °C for 5m. We based the number of cycles used for each library on the pre-PCR library concentrations. We purified completed PCR reactions using 1X SPRI, we re-hydrated libraries in 33 µL ddH2O, and we quantified 2 µL of each library using a Qubit fluorometer. We combined groups of eight libraries at equimolar ratios into enrichment pools having a final concentration of 147 ng/µL in 3.4 µL (500 ng total).

2. Target enrichment, and sequencing of UCEs.

We enriched libraries using an acanthomorph probe set targeting 1314 UCE loci and containing 2628 probes (Alfao et al. in prep), and we followed library enrichment procedures for the MYcroarray MYBaits kit ([Blumenstiel, et al. 2010](#_ENREF_1)), with three modifications: (1) we added 100 ng MYBaits to each reaction (a 1:5 dilution of the standard MYBaits concentration), (2) we added 500 ng custom blocking oligos designed against our custom sequence tags and using 10 inosines to block the 10 nucleotide index sequence, and (3) we used commercially available chicken Cot-1 DNA (Applied Genetics Laboratories) in place of the human Cot-1 provided by MYcroarray. We ran the hybridization reaction for 24 hours at 65 °C. Following hybridization we bound all pools to streptavidin beads (MyOne C1, Life Technologies) and washed bound libraries according to a standard target enrichment protocol ([Blumenstiel, et al. 2010](#_ENREF_1)). We removed the final aliquot of wash buffer following enrichment and allowed samples to dry for five minutes while sitting in a magnet stand. We removed residual buffer with sterile toothpicks. Then, we added 30 µL ddH20 to each sample and proceeded directly to PCR recovery while the enriched libraries were still bound to streptavidin beads ([Fisher, et al. 2011](#_ENREF_7)). The streptavidin beads do not inhibit PCR and with-bead PCR recovery of enriched libraries is a faster and easier procedure. We combined 15 µL of streptavidin bead-bound, enriched library in water with 25 µL HiFi HotStart Taq (Kapa Biosystems), 5 µL of Illumina TruSeq primer mix (5 µM each), and 5 µL of ddH2O. We PCR-recovered each library using the following thermal profile: 98 °C for 45s; 16 cycles of 98 °C for 15s, 60 °C for 30s, 72 °C for 60s; and a final extension of 72 °C for 5m. We placed the resulting PCR reactions in a magnet stand and removed the supernatant to separate the PCR-recovered, enriched DNA (supernatant) from the streptavidin beads. We subsequently purified the enriched DNA for each pool using 1X SPRI, and we re-hydrated enriched DNA in 33 µL ddH2O. We quantified 2 µL of each enriched pool using a Qubit fluorometer, and we diluted enriched pools to 2.5 ng/µL in 10 mM Tris-HCl prior to library size determined and qPCR quantification. We estimated library size by running diluted, enriched pools on an Agilent Bioanalyzer, and we quantified diluted, enriched pools using qPCR quantification (Kapa Biosytems). Based on the size-adjusted concentrations estimated by qPCR for each enriched pool, we combined 7 diluted, enriched pools of 8 samples from this study with 10 diluted enriched pools from a separate study (192 samples in total) to create an equimolar pool-of-pooled-libraries at 10 nM concentration. We sequenced 10 pmol of this mixture using 1.5 lanes of PE143 sequencing on an Illumina HiSeq in RapidRun mode (UCLA Neuroscience Genomics Core). Raw read data are archived in the NCBI Sequence Repository Archive (SRA), and assembled contigs archived on GenBank (accession information to be included before publication, see Additional information, below).

3. Analysis of captured sequence data

We demultiplexed samples and converted BCL data to FASTQ using *bcl2fastq* (v1.8.4) with no error correction, and we combined samples from different lanes using *merge_multiple_gzip_files.py*, which is part of the *phyluce* ([Faircloth 2015](#_ENREF_5)) software package (version 1.4). We trimmed reads to remove adapter contamination and low quality bases using a parallel wrapper (https://github.com/faircloth-lab/illumiprocessor) around *trimmomatic* ([Bolger, et al. 2014](#_ENREF_2)). Following read trimming, we computed summary statistics on the data using *get_fastq_stats.py* from *phyluce*. We assembled cleaned reads using a parallel wrapper around *trinity* (trinityrnaseq-r2013-02-25; assemblo_trinity.py; [Grabherr, et al. 2011](#_ENREF_8); [Marcais and Kingsford 2011](#_ENREF_13)) that applied the *--min-kmer-coverage=2* option during the assembly process. We computed coverage across assembled contigs using a program (*get_trinity_coverage.py*) that re-aligns the trimmed sequence reads to each set of assembled contigs using *bwa-mem* ([Li 2013](#_ENREF_9)), cleans the resulting BAM files using *picard* (1.99; http://picard.sourceforge.net/), adds read-group (RG) information to each library using *picard*, indexes the resulting BAM file using *samtools* ([Li, et al. 2009](#_ENREF_10)), and calculates coverage at each base of each assembled contig using *GATK* [2.7.2] ([DePristo, et al. 2011](#_ENREF_4); [McKenna, et al. 2010](#_ENREF_14); [Van der Auwera, et al. 2002](#_ENREF_18)).

To identify assembled contigs representing enriched UCE loci, we aligned species-specific contig assemblies to a FASTA file of all enrichment baits using *match_contigs_to_loci.py*. This program implements the matching process using LASTZ and ensures that matches are 80% identical over 80% of their length. This program also screens and removes apparent duplicate contigs or contigs that are hit by baits targeting more than one UCE locus. After screening and removing non-target and duplicated or misassembled contigs, the program creates a relational database containing several tables that map the contig names generated by the assembler to the names of each corresponding UCE locus across all taxa. We created a file containing the names of 55 enriched taxa from which we collected data (Supplementary Table 1), and we input this list to an additional program (*get_match_counts.py*) that queries the relational database to generate two lists: one containing those loci having data for all taxa (a “complete” data matrix) and another containing all loci having data for any taxon (an “incomplete” data matrix). We input these lists of loci to an additional program (*get_fastas_from_match_counts.py*) to create separate monolithic FASTA files matching the locus lists for complete and incomplete UCE data matrices. We exploded each monolithic FASTA by locus and we aligned sequence data for loci containing more than four taxa using *SATé* ([Liu, et al. 2009](#_ENREF_11); [Liu, et al. 2012](#_ENREF_12); [Sukumaran and Holder 2010](#_ENREF_16); [Yu, et al. 2014](#_ENREF_19)). Following *SATé* alignment, we removed the locus names from all alignments (*remove_locus_name_from_nexus_lines.py*), and trimmed resulting alignments using *gblocks* ([Castresana 2000](#_ENREF_3); [Talavera and Castresana 2007](#_ENREF_17)). For the complete UCE data matrix, we computed alignment statistics and the number of informative sites across all alignments using *get_align_summary_data.py* and *get_informative_sites.py*, we concatenated the resulting alignments into a PHYLIP-formatted supermatrix (*format_nexus_files_for_raxml.py*), and we analyzed the data as described below. For the incomplete matrix, we filtered the entire set of aligned loci to create two different incomplete matrices – a 95% complete matrix (alignments contained ≥ 52 of 55 taxa) and a 75% complete matrix (alignments contained ≥ 41 of 55 taxa). Following alignment filtering, we computed alignment statistics and the number of informative sites across all alignments, and we concatenated the resulting alignments, as described above.

**4. Additional information**

The bait set and enrichment protocols used here are available from http://ultraconserved.org (CC-BY-3.0) and Dryad (doi: xxxx/yyy.yyy). Computer programs used throughout this study are available from https://github.com/faircloth-lab/illumiprocessor and http://github.com/faircloth-lab/phyluce under an open-source, BSD-style license. Sequence data generated for this manuscript are available from the NCBI Sequence Read Archive (SRA PRJNAXXXX) and contig assemblies representing UCE loci are available from Genbank (## ZZZ-QQQ). Additional data including the probe set design file, sequence alignments, alignment supermatrices, configuration and output files, and inferred trees are available from Dryad (doi: xxxx/yyy.yyy).

**Supplementary Table S1.** Specimens used for DNA sequencing.

| **Species** | **Family** | **Catalogue Information** |
| --- | --- | --- |
| *Lampris guttatus* | Lampridae | YFTC 18113 |
| *Myripristis violacea* | Holocentridae | YFTC 12630 |
| *Kurtus gulliveri* | Kurtidae | YFTC 18514 |
| *Fibramia lateralis* | Apogonidae | YFTC 12659 |
| *Scomber scombrus* | Scombridae | YFTC 13855 |
| *Pseudupeneus maculatus* | Mullidae | YFTC 19662 |
| *Syngnathus fuscus* | Syngnathidae | YFTC 19982 |
| *Acanthurus bahianus* | Acanthuridae | YFTC 22882 |
| *Sphyraena putnamae* | Sphyraenidae | KU 6785 |
| *Sphyraena sphyraena* | Sphyraenidae | FMNH LS499 |
| *Lates calcarifer* | Latidae | MAGNT 92 |
| *Centropomus medius* | Centropomidae | KU:KUIT:8498 |
| *Toxotes jaculatrix* | Toxotidae | LSUMZ-F 5166 |
| *Leptobrama muelleri* | Leptobramidae | FMNH 119722 |
| *Mene maculata* | Menidae | G. Lecointre, B153 |
| *Xiphias gladius* | Xiphiidae | FMNH LS787 |
| *Tetrapturus angustirostris* | Istiophoridae | SIO 05-31 |
| *Istiophorus platypterus* | Istiophoridae | KU:KUIT:5428 |
| *Echeneis neucratoides* | Echeneidae | KU:KUIT:41346 |
| *Coryphaena hippurus* | Coryphaenidae | KU:KUIT:7212 |
| *Rachycentron canadum* | Rachycentridae | KU:KUIT:3521 |
| *Scomberoides commersonnianus* | Carangidae | KU:KUIT:8988 |
| *Trachinotus ovatus* | Carangidae | YFTC 19667 |
| *Trachinotus blochii* | Carangidae | KU:KUIT:6793 |
| *Seriola zonata* | Carangidae | KU:KUIT:1188 |
| *Decapterus maruadsi* | Carangidae | KU:KUIT:8984 |
| *Trachurus trachurus* | Carangidae | KU:KUIT:8077 |
| *Alectis indica* | Carangidae | KU:KUIT:8966 |
| *Megalaspis cordyla* | Carangidae | KU:KUIT:4705 |
| *Caranx melampygus* | Carangidae | KU:KUIT:5657 |
| *Alepes kleinii* | Carangidae | KU:KUIT:8986 |
| *Chloroscombrus orqueta* | Carangidae | KU:KUIT:8495 |
| *Polydactylus sexfilis* | Polynemidae | KU:KUIT:6829 |
| *Eleutheronema tetradactylum* | Polynmeidae | H. Larson |
| *Psettodes erumei* | Psettodidae | LSUMZ-F 5295 |
| *Citharus linguatula* | Citharidae | FMNH LS596 |
| *Citharoides macrolepis* | Citharidae | KU:KUIT:2468 |
| *Neoachiropsetta milfordi* | Achiropsettidae | FMNH LS715 |
| *Mancopsetta maculata* | Achiropsettidae | FNMH LS714 |
| *Gymnachirus melas* | Achiridae | KU:KUIT:5187 |
| *Trinectes maculatus* | Achiridae | KU:KUIT:1501 |
| *Samariscus xenicus* | Samaridae | KU:KUIT:2484 |
| *Poecilopsetta plinthus* | Poecilopsettidae | KU:KUIT:2473 |
| *Symphurus plagiusa* | Cynoglossidae | AMNH Uncat E474 |
| *Aseraggodes xenicus* | Cynoglossidae | KU:KUIT:5719 |
| *Scophthalmus rhombus* | Scophthalmidae | KU:KUIT:5416 |
| *Hypsopsetta guttulata* | Pleuronectidae | SIO 01-179 |
| *Hippoglossina oblonga* | Pleuronectidae | KU:KUIT:1493 |
| *Paralichthys albigutta* | Paralichthyidae | AMNH Uncat E172 |
| *Bothus pantherinus* | Bothidae | KU:KUIT:5642 |
| *Crossorhombus kobensis* | Bothidae | KU:KUIT:2485 |
| *Etropus cyclosquamus* | Paralichthyidae | KU:KUIT:5241 |
| *Cyclopsetta fimbriata* | Paralichthyidae | KU:KUIT:3986 |

**Institutional abbreviations:** AMNH, American Museum of Natural History; FMNH, Field Museum; KU, Kansas University Biodiversity Institute; LSUMZ, Lousiana State Museum of Natural Science; MAGNT, Museum and Gallery of the Northern Territory; KU, Kansas University Biodiversity Institute; SIO, Scripps Institution of Oceanography; YFTC, Yale University Fish Tissue Collection.

**Supplementary Table S2.** Comparative materials of Carangimorpha examined.

| **Species** | **Family** | **Catalogue Information** |
| --- | --- | --- |
| *Citharus lingulata* | Citharidae | FMNH LS596 |
| *Echeneis neucratoides* | Echeneidae | KU 41346. |
| *Eleutheronema tetradactylum* | Polynemidae | H. Larson |
| *Hypopsetta guttulata* | Pleuronectidae | SIO 01-179 |
| *Lates calcarifer* | Centropomidae | MAGNT 92 |
| *Leptobrama muelleri* | Leptobramidae | FMNH 119722 |
| *Mancopsetta maculata* | Achriopsettidae | FMNH LS714 |
| *Neoachiropsetta milfordi* | Achriopsettidae | FMNH LS715 |
| *Mene maculata* | Menidae | G. Lecointre, B153 |
| *Paralichthys albigutta* | Paralichthyidae | AMNH uncat. E172 |
| *Sphyraena sphyraena* | Sphyraenidae | FMNH LS499 |
| *Symphurus plagiusa* | Cynoglossus | AMNH uncat. E474 |
| *Tetrapturus angustirostris* | Istiophoridae | SIO 05-31 |
| *Trinectes maculatus* | Achiridae | KU 1501 |

**Institutional abbreviations:** AMNH, American Museum of Natural History; FMNH, Field Museum; MAGNT, Museum and Gallery of the Northern Territory; KU, Kansas University Biodiversity Institute; SIO, Scripps Institution of Oceanography.

**Supplementary Table S3**. Divergence date estimates and 95% Highest Posterior Densities (HPD) for selected Carangimorpha nodes, generated using BEAST (four analyses using different random samples of 75 UCE loci) and PAML (596 UCE loci). Nodes denoted with asterisk (*) represent fossil calibration points.

| **Node** | **BEAST 1** | **BEAST 2** | **BEAST 3** | **BEAST 4** | **PAML** |
| --- | --- | --- | --- | --- | --- |
| Carangimorpha | **71.05** [63.44-80.86] | **71.52** [63.58-81.41] | **72.13** [63.5-83.36] | **70.43** [62.58-80.17] | **70.68** [67.85-73.82] |
| Carangimorpha (excluding Sphyraenidae) | **70.04** [62.73-79.47] | **69.45** [62.17-76.17] | **70.45** [62.4-481] | **69.02** [59.63-72.99] | **70.34**[67.57-73.55] |
| Carangimorpha (excluding Sphyraenidae, Centropomidae) | **67.50** [60.98-75.82] | **67.16** [60.84-74.95] | **68.12** [61.2-77.79] | **66.7** [60.36-74.46] | **69.44** [66.64-72.59] |
| Sphyraenidae | **27.78** [4.68-60.81] | **29.81** [7.09-63.19] | **32.96** [9.25-64.51] | **27.14** [5.24-62.85] | **33.04** [18.39-52.13] |
| Carangoidei | **53.41** [50.01-59.73] | **53.73** [50.18-59.55] | **52.82** [49.84-58.23] | **52.81** [49.84-58.48] | **52.09** [50.26-54.13] |
| Carangini | **20.88** [8.37-38.29] | **22.95** [11.07-38.96] | **23.71** [11.07-40.34] | **20.68** [8.89-37.96] | **15.61** [12.3-19.12] |
| Xiphioidei | **38.09** [12.96-58.83] | **41.11** [17.76-59.28] | **36.48** [13.97-57.61] | **37.45** [18.38-59.36] | **52.29** [44.33-60.74] |
| Menidae and Xiphioidea* | **60.61** [56.51-66.56] | **60.2** [56.5-65.52] | **60.58** [56.54-66.81] | **60.35** [56.57-65.93] | **66.16** [63.02-69.49] |
| Polynemidae | **10.0** [1.04-31.57] | **12.55** [1.41-36.03] | **13.65** [1.93-45.75] | **13.19** [2.12-38.81] | **9.33** [5.46-13.72] |
| Pleuronectoidei and Psettodidae | **61.3** [54.28-69.45] | **62.25** [55.14-70.12] | **65.91** [54.61-72.12] | **61.21** [54.63-68.97] | **66.89** [64.14-69.84] |
| Pleuronectoidei | **56.83** [50.68-64.45] | **57.47** [50.92-64.78] | **62.61** [50.62-66.13] | **56.75** [50.95-63.70] | **61.82** [59.52-64.36] |
| Polynemidae and Pleuronectoidei* | **64.2** [57.28-72.62] | **65.21** [58.49-73.34] | **65.91** [58.13-75.93] | **64.37** [57.76-72.30] | **68.75** [65.98-71.86] |
| Centropomidae and Latidae* | **56.3** [50.0-66.69] | **60.36** [50.48-71.36] | **57.38** [50.1-69.12] | **58.27** [50.25-69.26] | **68.83** [65.86-72.14] |
| Toxotidae and Leptobramidae | **40.25** [7.04-63.79] | **39.56** [9.50-62.70] | **41.79** [10.03-64.66] | **37.6** [6.96-62.70] | **62.99** [56.4-68.48] |
| Rachycentridae and Coryphaenidae | **20.19** [3.93-36.32] | **23.29** [6.06-38.16] | **17.94** [3.47-35.17] | **20.67** [2.82-37.35] | **22.48** [14.11-30.90] |
| Echeneoidei* | **38.88** [33.01-46.55] | **38.66** [33.12-46.21] | **38.78** [33.06-46.31] | **39.22** [33.23-47.21] | **34.84** [29.62-41.66] |

References

Blumenstiel B, Cibulskis K, Fisher S, DeFelice M, Barry A, Fennell T, Abreu J, Minie B, Costello M, Young G, Maquire J, Kernytsky A, Melnikov A, Rogov P, Gnirke A, Gabriel S 2010. Targeted exon sequencing by in-solution hybrid selection. Curr Protoc Hum Genet Chapter 18: Unit 18 14. doi: 10.1002/0471142905.hg1804s66

Bolger AM, Lohse M, Usadel B 2014. Trimmomatic: a flexible trimmer for Illumina sequence data. Bioinformatics 30: 2114-2120. doi: 10.1093/bioinformatics/btu170

Castresana J 2000. Selection of conserved blocks from multiple alignments for their use in phylogenetic analysis. Molecular Biology and Evolution 17: 540-552.

DePristo MA, Banks E, Poplin R, Garimella KV, Maguire JR, Hartl C, Philippakis AA, del Angel G, Rivas MA, Hanna M, McKenna A, Fennell TJ, Kernytsky AM, Sivachenko AY, Cibulskis K, Gabriel SB, Altshuler D, Daly MJ 2011. A framework for variation discovery and genotyping using next-generation DNA sequencing data. Nature Genetics 43: 491-498. doi: 10.1038/ng.806

Faircloth BC 2015. PHYLUCE is a software package for the analysis of conserved genomic loci. Bioinformatics. doi: 10.1093/bioinformatics/btv646

Faircloth BC, Glenn TC 2012. Not all sequence tags are created equal: designing and validating sequence identification tags robust to indels. PLOS One 7: e42543. doi: 10.1371/journal.pone.0042543

Fisher S, Barry A, Abreu J, Minie B, Nolan J, Delorey TM, Young G, Fennell TJ, Allen A, Ambrogio L, Berlin AM, Blumenstiel B, Cibulskis K, Friedrich D, Johnson R, Juhn F, Reilly B, Shammas R, Stalker J, Sykes SM, Thompson J, Walsh J, Zimmer A, Zwirko Z, Gabriel S, Nicol R, Nusbaum C 2011. A scalable, fully automated process for construction of sequence-ready human exome targeted capture libraries. Genome Biol 12: R1. doi: 10.1186/gb-2011-12-1-r1

Grabherr MG, Haas BJ, Yassour M, Levin JZ, Thompson DA, Amit I, Adiconis X, Fan L, Raychowdhury R, Zeng Q, Chen Z, Mauceli E, Hacohen N, Gnirke A, Rhind N, di Palma F, Birren BW, Nusbaum C, Lindblad-Toh K, Friedman N, Regev A 2011. Full-length transcriptome assembly from RNA-Seq data without a reference genome. Nat Biotechnol 29: 644-652. doi: 10.1038/nbt.1883

Li H 2013. Aligning sequence reads, clone sequences, and assembly contigs with BWA-MEM. arXiv.org.

Li H, Handsaker B, Wysoker A, Fennell T, Ruan J, Homer N, Marth G, Abecasis G, Durbin R, Genome Project Data Processing S 2009. The Sequence Alignment/Map format and SAMtools. Bioinformatics 25: 2078-2079. doi: 10.1093/bioinformatics/btp352

Liu K, Raghavan S, Nelesen S, Linder CR, Warnow T 2009. Rapid and accurate large-scale coestimation of sequence alignments and phylogenetic trees. Science 324: 1561-1564. doi: 10.1126/science.1171243

Liu K, Warnow TJ, Holder MT, Nelesen SM, Yu J, Stamatakis AP, Linder CR 2012. SATe-II: very fast and accurate simultaneous estimation of multiple sequence alignments and phylogenetic trees. Syst Biol 61: 90-106. doi: 10.1093/sysbio/syr095

Marcais G, Kingsford C 2011. A fast, lock-free approach for efficient parallel counting of occurrences of k-mers. Bioinformatics 27: 764-770. doi: 10.1093/bioinformatics/btr011

McKenna A, Hanna M, Banks E, Sivachenko A, Cibulskis K, Kernytsky A, Garimella K, Altshuler D, Gabriel S, Daly M, DePristo MA 2010. The Genome Analysis Toolkit: a MapReduce framework for analyzing next-generation DNA sequencing data. Genome Res 20: 1297-1303. doi: 10.1101/gr.107524.110

Rohland N, Reich D 2012. Cost-effective, high-throughput DNA sequencing libraries for multiplexed target capture. Genome Res 22: 939-946. doi: 10.1101/gr.128124.111

Sukumaran J, Holder MT 2010. DendroPy: a Python library for phylogenetic computing. Bioinformatics 26: 1569-1571. doi: 10.1093/bioinformatics/btq228

Talavera G, Castresana J 2007. Improvement of phylogenies after removing divergent and ambiguously aligned blocks from protein sequence alignments. Syst Biol 56: 564-577. doi: 10.1080/10635150701472164

Van der Auwera GA, Carneiro MO, Hartl C, Poplin R, Ddel Angel G, Levy-Moonshine A, Jordan T, Shakir K, Roazen D, Thibault J, Banks E, Garimella KV, Altshuler D, Gabriel S, DePristo MA. 2002. From FastQ data to high-confidence variant calls: The genome analysis toolkit best practices pipeline. In. Current Protocols in Bioinformatics: John Wiley & Sons, Ltd.

Yu J, Holder MT, Sukumaran J, Mirarab S, Oaks J. 2014. SATé version 2.2.1: From <http://phylo.bio.ku.edu/software/sate/sate.html> 15 February 2013.

**Supplementary Figure Legends**

**Supplementary Figure S1.** Phylogenies for Carangimorpha inferred from partitioned analysis of concatenated data. Identical topologies were inferred by Bayesian and maximum likelihood analyses of (*a*) 1014 loci (75% complete matrix) (*b*) 596 loci (95% complete matrix) and (*c*) 97 loci (100% complete data matrix). Nodes indicated with solid black dots received maximum likelihood and Bayesian posterior probabilities of 100% and 1.0, respectively. All other nodes (indicated with white dots) have maximum likelihood and Bayesian posterior probabilities listed adjacent to the node. Flatfishes are indicated in red.

**Supplementary Figure S2.** Phylogenies for Carangimorpha inferred through coalescent species tree analysis. (*a*) 75% complete data matrix of 1014 loci. (*b*) 95% complete data matrix of 596 loci. Maximum likelihood bootstrap support values indicated next to nodes. Flatfishes are indicated in red.

**Supplementary Figure S3.** (*a*) Primary concordance tree and (*b*) population tree inferred from Bayesian concordance analysis in BUCKy. Concordance factors indicated adjacent to nodes.

**Supplementary Figure S4.** (*a*) PAML-inferred time-calibrated phylogeny of Carangimorpha based on 596 UCE loci. Histograms in *b*, *c*, and *d* are the same as those presented in Figure 1, depicting BEAST-inferred posterior distributions of branch lengths subtending the origin of bodyplans for (*b*) billfishes (Xiphioidei) and moonfishes (Menidae); (*c*) remoras (Echeneidae); and (*d*) flatfishes (Pleuronectiformes). The vertical red line in *b, c,* and *d* represents the PAML-estimated time for bodyplan origin, and its value, *l*, in millions of years, is also indicated next to each histogram. Red dots denote fossil-calibrated nodes.

**Supplementary Figure S1.**


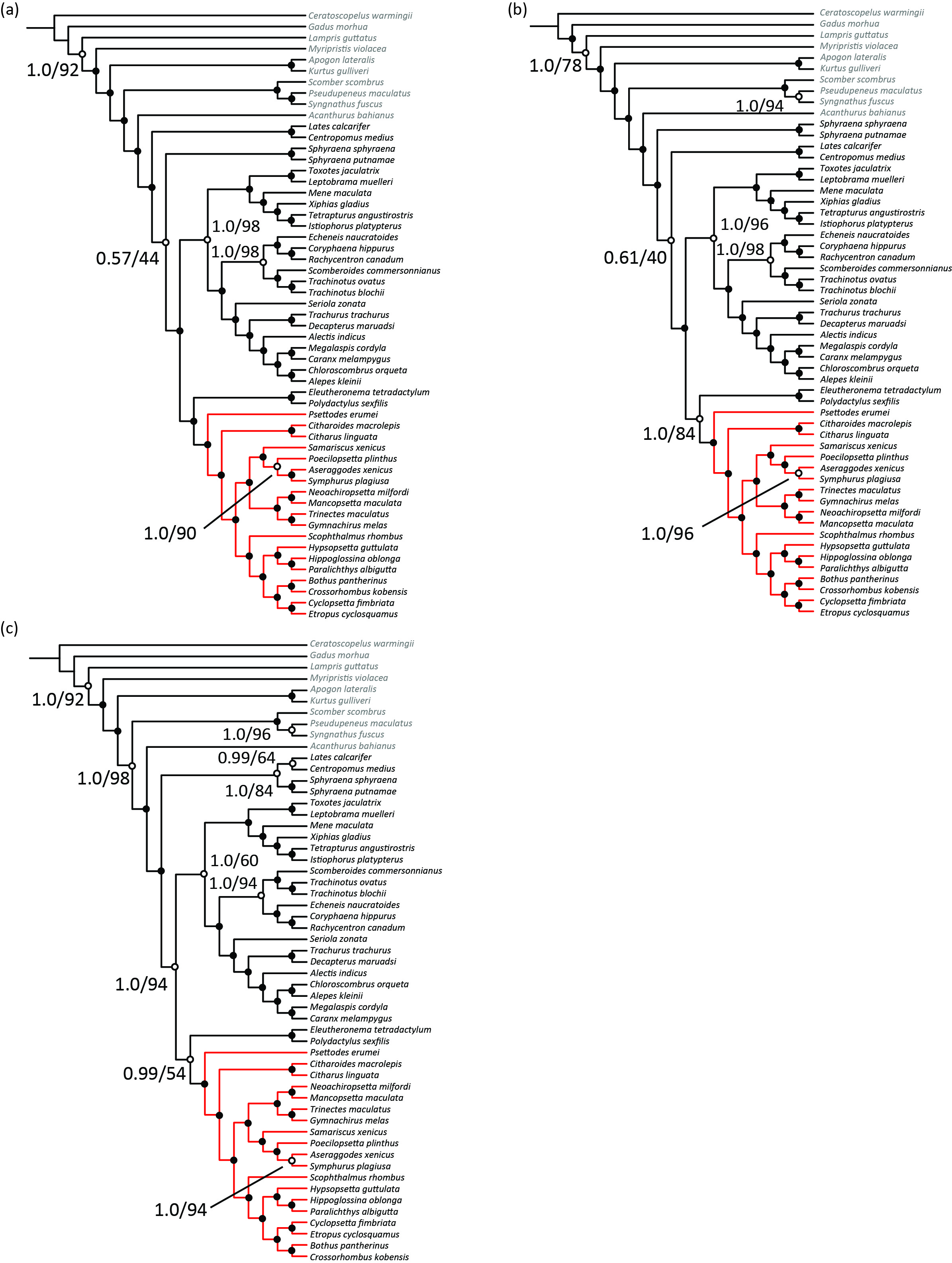


**Supplementary Figure S2.**


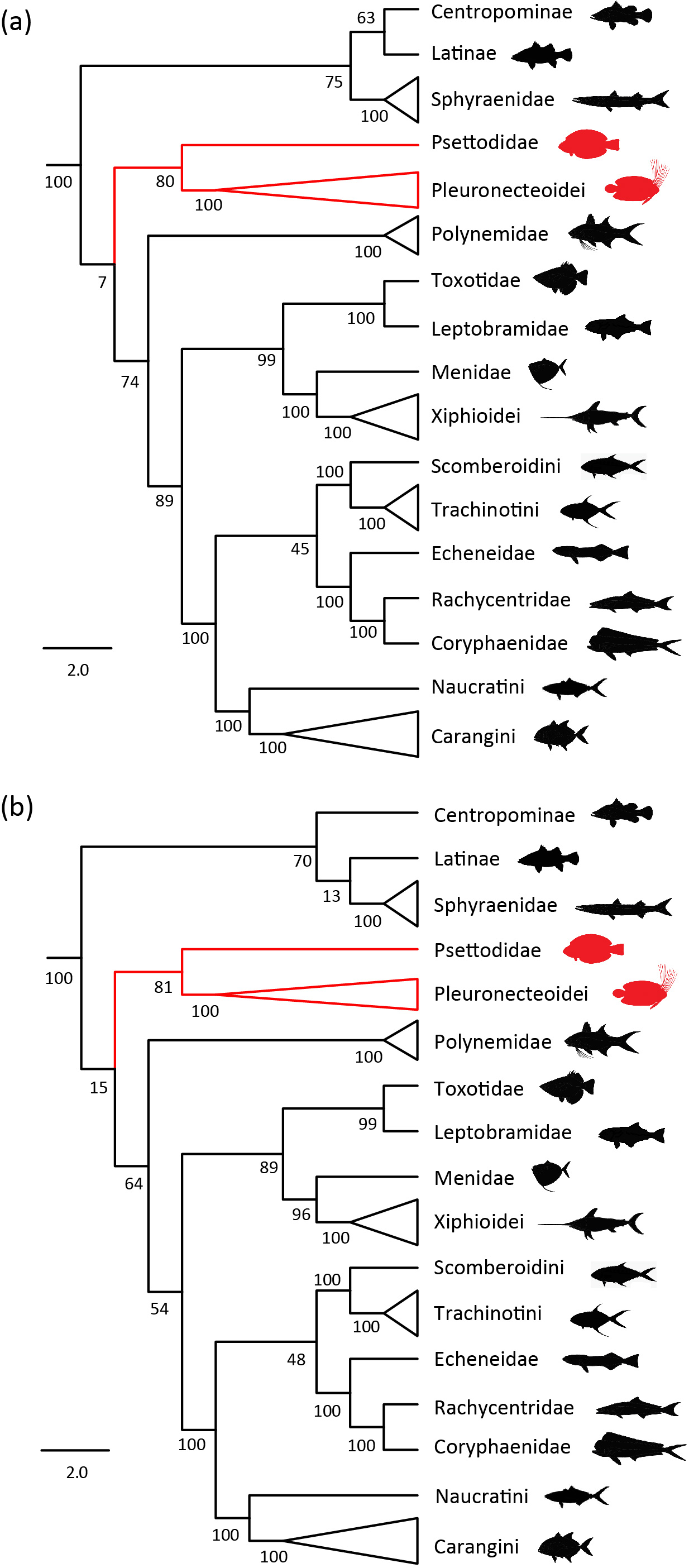


**Supplementary Figure S3**.


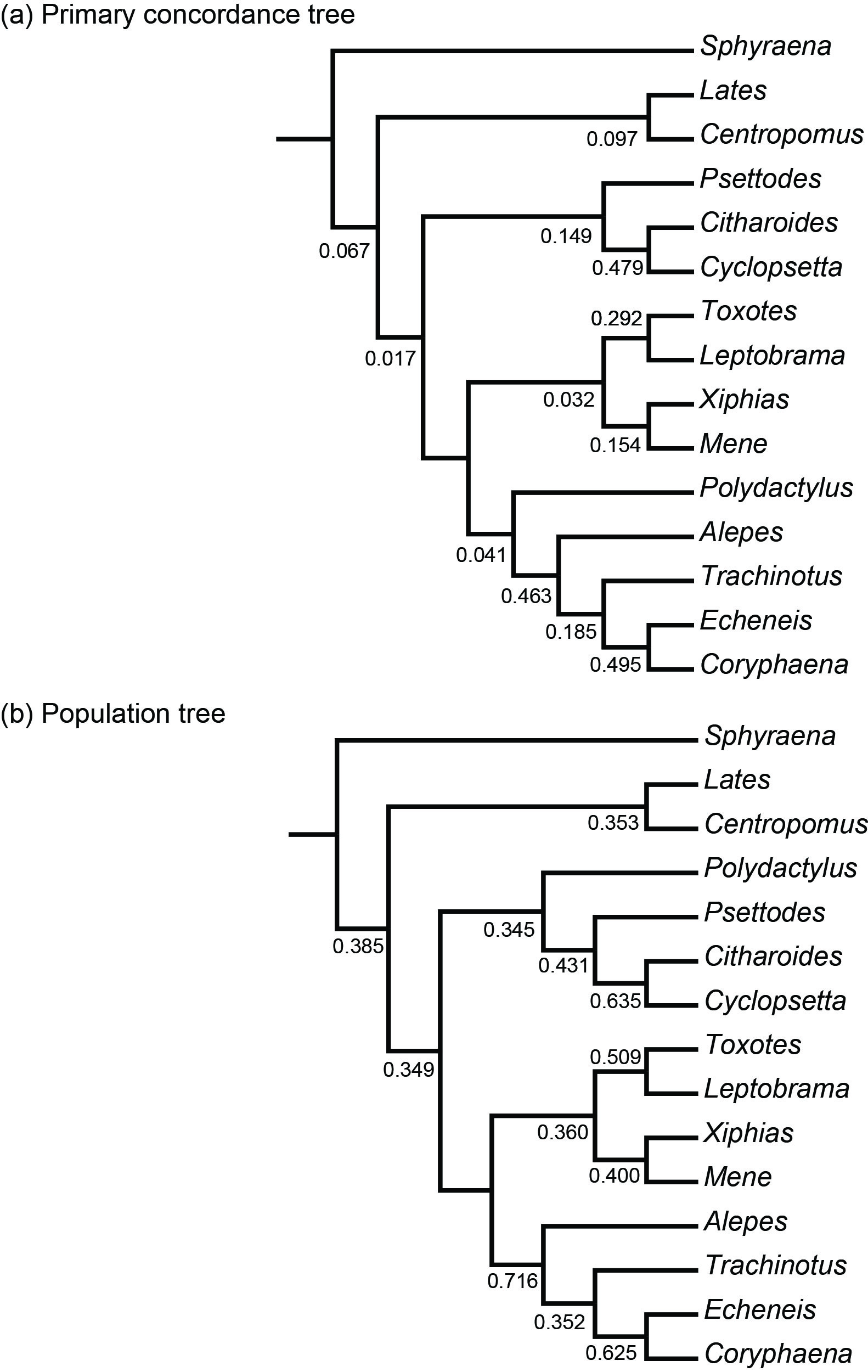


**Supplementary Figure S4.**

**
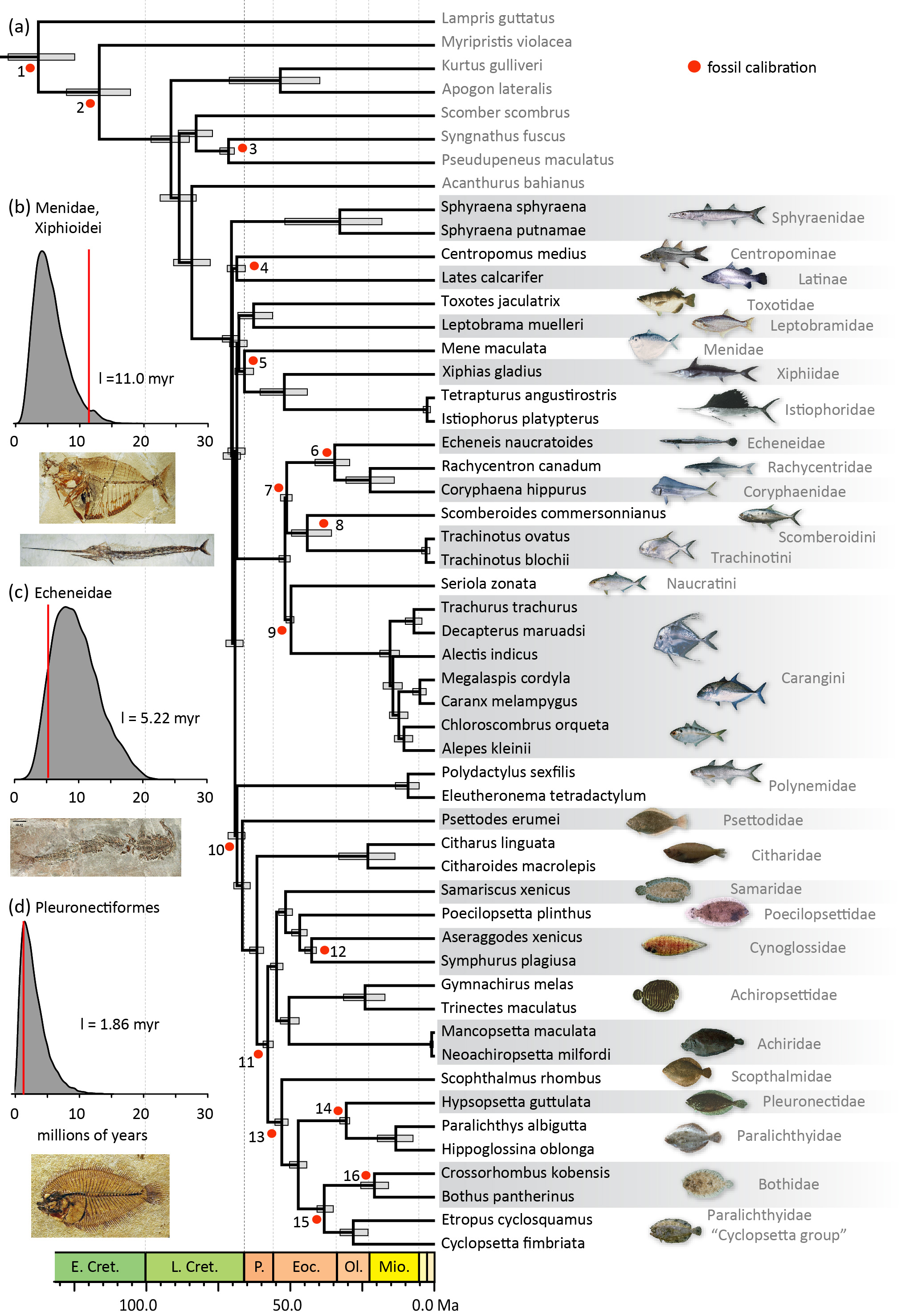
**
