# Supplementary material for: Phylogenomic analysis of carangimorph fishes reveals flatfish asymmetry arose in a blink of the evolutionary eye
Source: BMC Evol Biol. 2016 Oct 21;16:224. doi: 10.1186/s12862-016-0786-x (PMC5073739; doi:10.1186/s12862-016-0786-x)
Supplement: Additional file 2: — Descriptions of fossil calibrations used in divergence dating analyses, including details of age priors and the fossil data upon which they are based. (DOCX 92 kb) [file 12862_2016_786_MOESM2_ESM.docx]

**Outgroups to Acanthomorpha.** Our outgroup sequence is a modification of those applied by Friedman *et al*. [[1](#_ENREF_1)] (for Euteleosts) and Dornburg *et al*. [[2](#_ENREF_2)] (for early diverging crown teleosts, members of the stem, and non-teleost outgroups). Unlike Friedman *et al*. [[1](#_ENREF_1)], we have not included ctenothrissiforms, as ongoing work suggests that at least some of the taxa attributed to this group are not closely related to acanthomorphs. Aulopiformes (125 Ma, based on *Atolvorator longipectoralis*; see argumentation in [[1](#_ENREF_1)]); non-eurypterygian Euteleostei (Leptolepides haerteisi, 150.94 Ma; see argumentation in [[1](#_ENREF_1), [3](#_ENREF_3)]); Otocephala (*Tischlingerichthys viho*li, 150.94 Ma; see argumentation in [[3](#_ENREF_3)]); Elopomorpha (†*Anaethalion zapporum*, 151.2 Ma; see argumentation in [[2](#_ENREF_2), [3](#_ENREF_3)]); †Ichthyodectiformes (166.1 Ma, based on †*Occithrissops willsoni*; see argumentation in [[1](#_ENREF_1)]); †*Leptolepis coryphaenoides* (181.7 Ma; see argumentation in [[1](#_ENREF_1)]); †*Dorsetichthys bechei* (193.81 Ma; see argumentation for †*Pholidophorus bechei* in [[1](#_ENREF_1)]); †Pholidophoridae (221.0 Ma, based on †*Knerichthys bronni*; approximate age estimate for the top of the Carnian given by [[4](#_ENREF_4)]); †*Prohalecites porroi* (236.0; based on age estimate and associated uncertainty for the top of the Norian given by [[4](#_ENREF_4)]); Holostei (247.1 Ma, based on †*Watsonulus eugnathoides*; see argumentation in [[1](#_ENREF_1)]). As in [Friedman, Keck [5](#_ENREF_5)], we specify a hard upper bound for our calculations of 322.8 Ma based on the stem neopterygian †*Discoserra*. This yields the following sequence of outgroup ages (in Ma): 247.1, 236.0, 221.0, 193.81, 181.7, 166.1, 151.2, 150.94, 150.94, 125. This sequence of outgroups was appended to those given below for nodes, and used to calculate empirically informed 95% CIs and means for prior distributions on node ages. These were incorporated into our analysis of divergence times following the protocols outlined in the main text.

**Fossil-based minima and associated prior distributions.** Here we follow the best practices for the specification of fossil calibrations as outlined by Parham *et al.* [[6](#_ENREF_6)] and implemented in Benton *et al*. [[3](#_ENREF_3)] and elsewhere. Prior distributions were estimated using the outgroup sequences provided in conjuction with the analytical script provided as supplement to Friedman *et al*. [[1](#_ENREF_1)].

**Calibration 1**

**Node calibrated.** MRCA of *Lampris guttatus* and *Myripristis violacea*.

**Fossil taxon and specimen.** *Aipichthys minor*, MNHN HDJ 65, Museum national d’Histoire Naturelle, Paris.

**Minimum age.** 98.0 Ma.

**Phylogenetic justification.** *Aipichthys minor* is placed on the lampridiform stem by Davesne *et al.* [[7](#_ENREF_7)] in a parsimony analysis of 67 morphological characters.

**Age justification.** Age evidence for the fish beds at Hadjula is reviewed by elsewhere [[3](#_ENREF_3)], but key details are provided for completeness. This horizon is located below reported occurrence of *Mantelliceras mantelli*, which defines the first complete ammonite zone of the Late Cretaceous. The top of the *Mantelliceras mantelli* Zone is dated at 98.0 Ma [[8](#_ENREF_8)], from which we derive a minimum age for *Aipichthys*.

**Outgroup age sequence.** 247.1, 236.0, 221.0, 193.81, 181.7, 166.1, 151.2, 150.94, 150.94, 125, 98.0

**Estimated constraints on node age prior.** Mean: 119.1 Ma; 95% CI: 143.0 Ma.

**Calibration 2**

**Node calibrated.** MRCA of *Myripristis violacea* and *Kurtus gulliveri*.

**Fossil taxon and specimen.** *Stichocentrus liratus*, NHMUK PV P.47835 (holotype), The Natural History Museum, London, UK.

**Minimum age.** 98.0 Ma.

**Phylogenetic justification.** The penultimate anal-fin spine of *Stichocentrus* is enlarged, and represents a synapomorphy of holocentroids [[9](#_ENREF_9), [10](#_ENREF_10)].

**Age justification.** Age evidence for the fish beds at Hadjula is reviewed by elsewhere [[3](#_ENREF_3)], but key details are provided for completeness. This horizon is located below reported occurrence of *Mantelliceras mantelli*, which defines the first complete ammonite zone of the Late Cretaceous. The top of the *Mantelliceras mantelli* Zone is dated at 98.0 Ma [[8](#_ENREF_8)], from which we derive a minimum age for *Stichocentrus*.

**Outgroup age sequence.** 247.1, 236.0, 221.0, 193.81, 181.7, 166.1, 151.2, 150.94, 150.94, 125, 98.0, 98.0

**Estimated constraints on node age prior.** Mean: 108.7 Ma; 95% CI: 128.8 Ma.

**Calibration 3**

**Node calibrated.** MRCA of *Pseudupeneus maculatus* and *Syngnathus fuscus*.

**Fossil taxon and specimen.** *Gasterorhamphosus zuppichini*, MCSNV Na T 877 (holotype and only specimen), Museo Civico di Storia Naturale, Verona, Italy.

**Minimum age.** 69.71 Ma.

**Phylogenetic justification.** Near *et al.* provided justification for a sygnathiform interpretation of *Gasterorhamphosus* [[11](#_ENREF_11)], citing the following features: absence of anal-fin spine, enlarged dorsal-fin spine with serrated posterior margin, elongated tubular snout, absence of pleural ribs, enlarged posterodorsal process of cleithrum, rod-like anteroventral process of coracoids and simple pectoral rays.

**Age justification.** Medizza and Sorbini (1980) provide a list of calcareous nannofossil species recovered from the fish-bearing layers, the most biostratigraphically relevant of which is *Uniplanarus trifidus* (reported as *Quadrum trifidum*). The first appearance of this species marks the beginning of Calcareous Nannoplankton Zone CC23, and it makes its last appearance in the middle of CC24. The top of CC24 is roughly equivalent to the top of the *Baculites clinolobatus* Ammonite Zone of the Western Interior Seaway, which contains a bentonite horizon dated as 70.08 Ma ± 0.37 Myr [[8](#_ENREF_8)]. It is from this value that we derive our minimum age estimate of 69.71 Ma.

**Outgroup age sequence.** 247.1, 236.0, 221.0, 193.81, 181.7, 166.1, 151.2, 150.94, 150.94, 125, 98.0, 98.0, 69.71, 69.71.

**Estimated constraints on node age prior.** Mean: 79.6 Ma; 95% CI: 98.1 Ma.

**Calibration 4**

**Node calibrated.** MRCA of *Lates calcarifer* and *Centropomus medius*.

**Fossil taxon and specimen.** *Eolates gracilis*, MNHN BOL 61, 62 (holotype), Museum national d’Histoire Naturelle, Paris.

**Minimum age.** 49 Ma.

**Phylogenetic justification.** *Eolates gracilis* is resolved as a sister lineage of *Lates* to the exclusion of *Centropomus* in a parsimony analysis of 29 morphological characters [[12](#_ENREF_12)].

**Age justification.** *Eolates gracilis* is known from the Pesciara locality of Bolca, Italy. A detailed review of the geology and age of this deposit is given by Papazzoni and colleagues [[13](#_ENREF_13)], but key details are summarized here. Pesciara can be constrained to the narrow interval of overlap between NP14 and SBZ11. This constrains the deposits to no younger than 49 Ma [[14](#_ENREF_14)], which we apply as a minimum age here.

**Outgroup age sequence.** 247.1, 236.0, 221.0, 193.81, 181.7, 166.1, 151.2, 150.94, 150.94, 125, 98.0, 98.0, 69.71, 69.71, 55.2, 49

**Estimated constraints on node age prior.** Mean: 58.3 Ma; 95% CI: 72.8 Ma.

**Calibration 5**

**Node calibrated.** MRCA of *Mene maculata* and *Xiphias gladius*.

**Fossil taxon and specimen.** *Mene purydi*, USNM 494403 (holotype and only specimen), National Museum of Natural History, Washington, DC, USA.

**Minimum age.** 55.20 Ma.

**Phylogenetic justification.** Friedman and Johnson [[15](#_ENREF_15)] interpret *Mene purdyi* as a menid on the based on a mixture of derived and general traits. *M. purdyi* shares three compelling synapomorphies with *Mene*: a cavernous vault formed by the frontals; an infraorbital series comprising numerous, small ossicles; and close application of the neural arches of the first two vertebrae.

**Outgroup age sequence.** Friedman and Johnson [[15](#_ENREF_15)] identified *Mene purdyi* as latest Thanetian-earliest Ypresian in age on the basis of a series of plantonic foraminifera collected from the matrix of the specimen consistent with foraminiferal zones P4c to P5. Specifically, the presence of *Morozovella velascoensis* provides an upper age limit of 55.20 Ma [[16](#_ENREF_16)].

**Calibration prior.** 247.1, 236.0, 221.0, 193.81, 181.7, 166.1, 151.2, 150.94, 150.94, 125, 98.0, 98.0, 69.71, 69.71, 55.2

**Estimated constraints on node age prior.** Mean: 67.5 Ma; 95% CI: 84.7 Ma.

**Notes.** Roughly contemporary specimens of *Mene* are known from the Stolleklint Clay of Denmark [[17](#_ENREF_17)] and the Danatinsk Formation of Turkmenistan [[18](#_ENREF_18), [19](#_ENREF_19)]. Available evidence constrains the minimum age of both deposits to earliest Eocene (see below in ‘Taxa not applied as calibrations, but employed in calculations of prior distributions’), so we adopt *Mene purdyi* as our calibration here.

**Calibration 6**

**Node calibrated.** MRCA of *Echeneis* cf. *naucratoides* and *Rachycentron canadum*.

**Fossil taxon and specimen.** Echeneidae undet., HLMD WT-36, Hessisches Landesmuseum, Darmstadt, Germany [[20](#_ENREF_20)].

**Minimum age.** 29.62 Ma.

**Phylogenetic justification.** Echeneidae undet. bears numerous synaomorphies of remoras, including a dorsal adhesion disc and expanded transverse processes of vertebrae [[21](#_ENREF_21), [22](#_ENREF_22)].

**Age justification.** The ‘fish shales’ of Grube Unterfeld (“Frauenweiler”) yielding Echenidae undet. lie within NP23 [[23](#_ENREF_23)]. The top of NP23 is dated to 29.62 Ma [[14](#_ENREF_14)], providing a minimum age of divergence between *Echeneis* cf. *naucratoides* and *Rachycentron canadum*.

**Outgroup age sequence.** 247.1, 236.0, 221.0, 193.81, 181.7, 166.1, 151.2, 150.94, 150.94, 125, 98.0, 98.0, 69.71, 69.71, 55.2, 54.17, 49.0, 49.0, 29.62

**Estimated constraints on node age prior.** Mean: 41.0 Ma; 95% CI: 51.9 Ma.

**Notes.** The Rupelian remora †*Opisthomyzon* bears an adhesion disc that is more primitive than those of Echeneidae undet. [[21](#_ENREF_21)]. Although the maximum age of this genus is given by radiometric dating of underlying sediments, its minimum age is not as well constrained [[21](#_ENREF_21)]; foraminiferans have not been reported from the deposits yielding †*Opisthomyzon* [[24](#_ENREF_24)]. Because the minimum age of Echeneidae undet. can be constrained through foraminiferal biostratigraphy (see above), we have selected this fossil as our calibration rather than the better known †*Opisthomyzon*.

**Calibration 7**

**Node calibrated.** MRCA of *Echeneis* cf. *naucratoides* and *Scomberoides commersonnianus*.

**Fossil taxon and specimen.** *Ductor vestenae*, MNHN BOL 96, Museum national d’Histoire Naturelle, Paris.

**Minimum age.** 49 Ma.

**Phylogenetic justification.** Based on a series of parsimony and Bayesian analyses of morphological plus molecular data and morphological data in isolation, Friedman *et al.* [[21](#_ENREF_21)] reported two alternative placements for *Ductor*: either as the sister taxon of crown Echeneoidei, or within crown Echeneoidei as sister to Rachycentridae plus Coryphaenidae. We adopt the former, interpretation here, as it represents a more conservative application of this fossil as a minimum.

**Age justification.** *Ductor vestenae* is known from the Pesciara locality of Bolca, Italy. A detailed review of the geology and age of this deposit is given by Papazzoni and colleagues [[13](#_ENREF_13)], but key details are summarized here. Pesciara can be constrained to the narrow interval of overlap between NP14 and SBZ11. This constrains the deposits to no younger than 49 Ma [[14](#_ENREF_14)], which we apply as a minimum age here.

**Outgroup age sequence.** 247.1, 236.0, 221.0, 193.81, 181.7, 166.1, 151.2, 150.94, 150.94, 125, 98.0, 98.0, 69.71, 69.71, 55.2, 54.17, 49.0, 49.0

**Estimated constraints on node age prior.** Mean: 52.2 Ma; 95% CI: 59.1 Ma.

**Calibration 8**

**Node calibrated.** MRCA of *Scomberoides commersonnianus* and *Trachnotus blochii*.

**Fossil taxon and specimen.** *Scomberoides spinosus*, PIN 485/72, Paleontological Institute of the Russian Academy of Sciences, Moscow, Russia.

**Minimum age.** 19.30 Ma.

**Phylogenetic justification.** *Scomberoides spinosus* shows two key hard-tissue synapomorphies of Scomberoidini [[25](#_ENREF_25), [26](#_ENREF_26)]: 26 vertebrae (other carangids generally have 24), and posterior fin rays of the dorsal and anal fin developed as finlets [[27](#_ENREF_27)].

**Age justification.** *Scomberoides spinosus* derives from sediments of the Upper Maikop at Chernaya Rechka, Caucasus [[19](#_ENREF_19)]. These deposits are placed within the Sakaraul regional stage, which is correlated with the upper part of Plantonic Foraminiferan Zone M2 [[28](#_ENREF_28)]. The top of M2 is dated as 19.30 Ma [[29](#_ENREF_29)], which we adopt as a minimum age for the divergence between *Scomberoides commersonnianus* and *Trachnotus blochii*.

**Outgroup age sequence.** 247.1, 236.0, 221.0, 193.81, 181.7, 166.1, 151.2, 150.94, 150.94, 125, 98.0, 98.0, 69.71, 69.71, 55.2, 54.17, 49.0, 49.0, 19.30

**Estimated constraints on node age prior.** Mean: 35.8 Ma; 95% CI: 50.9 Ma.

**Notes.** The age estimate provided here is a minor adjustment from previous applications of this calibration. Bannikov [[18](#_ENREF_18)] has interpreted the Eocene (Bartonian) *Quasioligoplites mirus* as a member of Scomberoidini, which, if correct, means that our proposed minimum is a substantial underestimate of the divergence between *Scomberoides commersonnianus* and *Trachinotus blochii*. Although *Quasioligoplites mirus* broadly resembles a scomberoidine, it does not clearly show diagnostic hard-tissue characters of this clade that are readily apparent in *Scomberoides spinosus* [[27](#_ENREF_27)], which we regard as a more conservative marker.

**Calibration 9**

**Node calibrated.** MRCA of *Seriola zonata* and *Chloroscombrus orqueta*.

**Fossil taxon and specimen.** *Eastmanalepes primaevus*, MCZ 50706 (holotype), Museum of Comparative Zoology, Harvard University, Cambridge, USA.

**Minimum age.** 49 Ma.

**Phylogenetic justification.** *Eastmanelepes* bears thickened scutes along its flank, representing a synapomorphy of Carangini within Carangidae [[25](#_ENREF_25), [26](#_ENREF_26)].

**Age justification.** *Eastmanelepes* is known from the Pesciara locality of Bolca, Italy. A detailed review of the geology and age of this deposit is given by Papazzoni and colleagues [[13](#_ENREF_13)], but key details are summarized here. Pesciara can be constrained to the narrow interval of overlap between NP14 and SBZ11. This constrains the deposits to no younger than 49 Ma [[14](#_ENREF_14)], which we apply as a minimum age here.

**Outgroup age sequence.** 247.1, 236.0, 221.0, 193.81, 181.7, 166.1, 151.2, 150.94, 150.94, 125, 98.0, 98.0, 69.71, 69.71, 55.2, 54.17, 49.0, 49.0

**Estimated constraints on node age prior.** Mean: 52.2 Ma; 95% CI: 59.1 Ma.

**Calibration 10**

**Node calibrated.** MRCA of *Psettodes erumei* and *Polydactylus sexfilis*.

**Fossil taxon and specimen.** *Heteronectes chaneti*, NHMW 1974.1639.24, 1974.1639.25 (holotype and only specimen), Naturhistorisches Museum, Vienna, Austria.

**Minimum age.** 49 Ma.

**Phylogenetic justification.** Friedman [[30](#_ENREF_30)] resolved *Heteronectes* as the deepest branch of the flatfish stem lineage in a parsimony analysis of 58 morphological characters.

**Age justification.** *Heteronectes* is known from the fish beds of Bolca, Italy, but whether it derives from the Pesciara or Monte Postale locality is unclear. A detailed review of the geology and age of this deposit is given by Papazzoni and colleagues [[13](#_ENREF_13)], but key details are summarized here. Pesciara can be constrained to the narrow interval of overlap between NP14 and SBZ11. This constrains the deposits to no younger than 49 Ma [[14](#_ENREF_14)], which we apply as a minimum age here.

**Outgroup age sequence.** 247.1, 236.0, 221.0, 193.81, 181.7, 166.1, 151.2, 150.94, 150.94, 125, 98.0, 98.0, 69.71, 69.71, 55.2, 49.0

**Estimated constraints on node age prior.** Mean: 58.3 Ma; 95% CI: 72.8 Ma.

**Calibration 11**

**Node calibrated.** MRCA of *Symphurus plagiusa* and *Bothus pantherinus*.

**Fossil taxon and specimen.** *Eobothus minimus*, BSP-AS-I-68, Bayerische Staatssammlung für Paläontologie un Historische Geologie, Munich, Germany.

**Minimum age.** 49 Ma.

**Phylogenetic justification.** Friedman [[30](#_ENREF_30)] resolved *Eobothus* as a crown pleuronectoid in a parsimony analysis of 58 morphological characters, but this genus provides a minimum age constraint for a more restricted flatfish clade. Chanet [[31](#_ENREF_31)] noted that *Eobothus* shares derived features of the caudal skeleton with Scophthalmidae, Bothidae, Pleuronectidae, Paralichthyidae and *Brachypleura*: fusion of hypurals 1 and 2 and 3 and 4, and the fusion of hypurals 3 and 4 to the first preural centrum. These traits appear to have evolved independently in *Brachypleura* and the remaining families [[32](#_ENREF_32)]. *Eobothus* shows several derived features common to the four ‘bothoid’ families (e.g., loss of pelvic-fin spine, anteriorly inclined neural spine of second abdominal vertebra) but not apparent in *Brachypleura* and other deeply diverging flatfish lineages. On this basis, we interpret *Eobothus* as a member of the clade comprising Scophthalmidae, Bothidae, Pleuronectidae and Paralichthyidae, but cannot resolve its placement relative to these lineages. As such, this taxon provides a minimum age for the divergence between *Symphurus plagiusa* and *Bothus pantherinus*.

**Age justification.** *Eobothus* is known from the Pesciara locality of Bolca, Italy. A detailed review of the geology and age of this deposit is given by Papazzoni and colleagues [[13](#_ENREF_13)], but key details are summarized here. Pesciara can be constrained to the narrow interval of overlap between NP14 and SBZ11. This constrains the deposits to no younger than 49 Ma [[14](#_ENREF_14)], which we apply as a minimum age here.

**Outgroup age sequence.** 247.1, 236.0, 221.0, 193.81, 181.7, 166.1, 151.2, 150.94, 150.94, 125, 98.0, 98.0, 69.71, 69.71, 55.2, 49.0, 49.0, 49.0

**Estimated constraints on node age prior.** Mean: 51.5 Ma; 95% CI: 58.3 Ma.

**Calibration 12**

**Node calibrated.** MRCA of *Symphurus plagiusa* and *Aseraggodes xenicus*.

**Fossil taxon and specimen.** *Eubuglossus eocenicus*, NHMUK PF P14485, the Natural History Museum, London, UK.

**Phylogenetic justification.** *Eubuglossus* shows clear synapomorphies of Pleuronectiformes and Pleuronectoidei [[33](#_ENREF_33)], as well as derived cranial features limited to soleoids [[34](#_ENREF_34), [35](#_ENREF_35)]. Chanet [[34](#_ENREF_34)] argues for placement within Soleidae on the basis of a medially (rather than dorsally) directed ascending process of the blind-side premaxilla [[35](#_ENREF_35)].

**Minimum age.** 41.2 Ma.

**Age justification.** *Eubuglossus* derives from the lower Mokkatam of Djebel Turah, southeast of Cairo, Egypt. The Mokkatam spans much of the Lutetian [[36](#_ENREF_36)], but the exact collection horizon within this sequence is not clear [[34](#_ENREF_34), [37](#_ENREF_37)]. The top of the Lutetian is dated approximately as 41.2 Ma [[14](#_ENREF_14)], providing a minimum age of divergence between *Symphurus plagiusa* and *Aseraggodes xenicus*.

**Outgroup age sequence.** 247.1, 236.0, 221.0, 193.81, 181.7, 166.1, 151.2, 150.94, 150.94, 125, 98.0, 98.0, 69.71, 69.71, 55.2, 49.0, 49.0, 49.0, 41.2

**Estimated constraints on node age prior.** Mean: 46.4 Ma; 95% CI: 52.8 Ma.

**Calibration 13**

**Node calibrated.** MRCA of *Scophthalmus rhombus* and *Bothus pantherinus*.

**Fossil taxon and specimen.** *Scophthalmus stamatini*, MSNPN 151 A-A’, Museum of Natural History, Piatra Neamt, Romania [[38](#_ENREF_38)].

**Phylogenetic justification.** *Scopthalamus stamatini* shows clear synapomorphies of Pleuronectiformes and Pleuronectoidei [[33](#_ENREF_33)], as well as derived features of the caudal-fin skeleton placing it within the ‘bothoid group’ [[33](#_ENREF_33), [38](#_ENREF_38)]. Baciu and Chanet [[38](#_ENREF_38)] justify placement within Scophthalmidae based on the presence of pelvic fins with long insertions that extend on to the urohyal. The presence of 11 precaudal vertebrae justifies attribution to *Scophthalmus* [[38](#_ENREF_38)].

**Minimum age.** 29.62 Ma.

**Age justification.** Nannoplankton from the fish-bearing shales of the lower dysodils exposed near Piatra Neamt place these beds within NP23 [[38](#_ENREF_38)]. The top of NP23 is dated to 29.62 Ma [[14](#_ENREF_14)], providing a minimum age of divergence between *Bothus pantherinus* and *Scophthalmus rhombus*.

**Outgroup age sequence.** 247.1, 236.0, 221.0, 193.81, 181.7, 166.1, 151.2, 150.94, 150.94, 125, 98.0, 98.0, 69.71, 69.71, 55.2, 49.0, 49.0, 49.0, 29.62

**Estimated constraints on node age prior.** Mean: 40.7 Ma; 95% CI: 51.3 Ma.

**Calibration 14**

**Node calibrated.** MRCA of *Paralichthys albiguttata* and *Hypopsetta guttulata*.

**Fossil taxon and specimen.** *Oligopleuronectes germanicus*, HLMD-WT 257, Hessisches Landesmuseum, Darmstadt, Germany [[23](#_ENREF_23)].

**Phylogenetic justification.** *Oligopleuronectes* shows clear synapomorphies of Pleuronectiformes and Pleuronectoidei [[33](#_ENREF_33)], as well as features of the caudal-fin endoskeleton placing it within a clade including Scophthalmidae, Paralichthyidae, Pleuronectidae and Bothidae [[23](#_ENREF_23), [32](#_ENREF_32)]. Defining characters of these individual families cannot be discerned in the type and only specimen, but Sakamoto *et al.* argue that placement within Pleuronectidae based on two lines of evidence [[23](#_ENREF_23)]. First, *Oligopleuronectes* is right-eyed, sharing this chirality with pleuronectids. This family is the only dextral lineage belonging this extended clade and is nested within it, suggesting this chirality is derived. Second, *Oligopleuronectes* bears a lateral process on the eye-side frontal, a derived feature shared with some pleuronectids [[39](#_ENREF_39)].

**Minimum age.** 29.62 Ma.

**Age justification.** The ‘fish shales’ yielding *Oligobothus* lie within NP23 [[23](#_ENREF_23)]. The top of NP23 is dated to 29.62 Ma [[14](#_ENREF_14)], providing a minimum age of divergence between *Paralichthys albiguttata* and *Hypopsetta guttulata*.

**Outgroup age sequence.** 247.1, 236.0, 221.0, 193.81, 181.7, 166.1, 151.2, 150.94, 150.94, 125, 98.0, 98.0, 69.71, 69.71, 55.2, 49.0, 49.0, 49.0, 29.62, 29.62

**Estimated constraints on node age prior.** Mean: 35.3 Ma; 95% CI: 45.8 Ma.

**Calibration 15**

**Node calibrated.** MRCA of *Bothus pantherinus* and *Cyclopsetta fimbriata*.

**Fossil taxon and specimen.** *Oligobothus pristinus*, MSNPN 189-500, Museum of Natural History, Piatra Neamt, Romania [[38](#_ENREF_38)].

**Phylogenetic justification.** *Oligobothus* shows clear synapomorphies of Pleuronectiformes and Pleuronectoidei [[33](#_ENREF_33)], as well as derived features of the caudal-fin skeleton placing it within the ‘bothoid group’ [[33](#_ENREF_33), [38](#_ENREF_38)]. Baciu and Chanet [[38](#_ENREF_38)] justify placement within Bothidae based on the presence of myorhabdoi, intermuscular bones with fimbrate proximal and distal ends [[40](#_ENREF_40)].

**Minumum age.** 29.62 Ma.

**Age justification.** Nannoplankton from the fish-bearing shales of the lower dysodils exposed near Piatra Neamt place these beds within NP23 [[38](#_ENREF_38)]. The top of NP23 is dated to 29.62 Ma [[14](#_ENREF_14)], providing a minimum age of divergence between *Bothus pantherinus* and *Cyclopsetta fimbriata*.

**Outgroup age sequence.** 247.1, 236.0, 221.0, 193.81, 181.7, 166.1, 151.2, 150.94, 150.94, 125, 98.0, 98.0, 69.71, 69.71, 55.2, 49.0, 49.0, 49.0, 29.62, 29.62, 29.62

**Estimated constraints on node age prior.** Mean: 32.6 Ma; 95% CI: 39.9 Ma.

**Calibration 16**

**Node calibrated.** MRCA of *Bothus pantherinus* and *Crossorhombus kobensis*.

**Fossil taxon and specimens.** *Bothus* sp., PIN 5073-12, 5073-13, 5073-14, Paleontological Institute of the Russian Academy of Sciences, Moscow [[41](#_ENREF_41)].

**Phylogenetic justification.** Carnevale *et al*. [[33](#_ENREF_33), [39-41](#_ENREF_39)] show that *Bothus* sp. from the north Caucasus shows clear synapomorphies of Pleuronectiformes, Pleuronectoidei, and Bothidae. They argue for more specific affinity with *Bothus* on the basis of robust, rectangular haemal spines that are diagnostic of the genus [[42](#_ENREF_42)].

**Minumum age.** 11.056 Ma.

Age justification. *Bothus* sp. derives from beds 11 and 12 of the Middle Tsurevsky Member of the Tsurevsky Formation along the bank of the Psheka River in western North Caucasus [[41](#_ENREF_41)]. Abundant remains of the bivalve *Abra reflexa* place this unit within the Volhynian regional stage[[43](#_ENREF_43), [44](#_ENREF_44)] of the Sarmatian sensu lato. This in turn is correlated with the Serravallian and earliest Tortonian of the international timescale [[28](#_ENREF_28), [29](#_ENREF_29)], and corresponds roughly to polarity chron C5r. The base of C5n.2n is dated as 11.056 Ma [[29](#_ENREF_29)], from which we derive a minimum age for the divergence between *Bothus pantherinus* and *Crossorhombus kobensis*.

**Outgroup age sequence.** 247.1, 236.0, 221.0, 193.81, 181.7, 166.1, 151.2, 150.94, 150.94, 125, 98.0, 98.0, 69.71, 69.71, 55.2, 49.0, 49.0, 49.0, 29.62, 29.62, 29.62, 11.056

**Estimated constraints on node age prior.** Mean: 21.9 Ma; 95% CI: 32.6 Ma.

**Taxa not applied as calibrations, but employed in calculations of prior distributions.** Carangimorphs have a rich fossil record, with many specimens that do not define calibration minima but can otherwise inform the estimation of prior distributions on node ages. The fossils listed below do so by being stratigraphically consistent (i.e., older than or coeval with the first fossil record of a more nested clade) with outgroup sequences derived from the above list of node calibrations.

**Total-group Carangiformes**

**Fossil taxon and specimen.** *Archaeus oblongus*, PIN 2179/94, Paleontological Institute of the Russian Academy of Sciences, Moscow, Russia.

**Minimum age.** 54.17 Ma.

**Phylogenetic justification.** NHMUK P23891 is identified as a carangid on the basis of a gap between the second and third anal-fin spines [[25](#_ENREF_25)].

**Age justification.** The Danatinsk Formation of Turkmenistan has been correlated with with widely distributed sapropels associated with the Paleocene-Eocene boundary in the Peri-Tethys. This deposit spans calcareous nannoplankton zones NP9-NP10 [[45](#_ENREF_45)], yielding a minimum age of 54.17 Ma [[16](#_ENREF_16)].

**Notes.** Roughly coeval remains of carangids are known from the Fur Formation of Denmark (NHMUK P23891; Natural History Museum, London, UK). This deposit spans calcareous nannoplankton zones NP10-NP11 [[46](#_ENREF_46)], yielding a minimum age of 53.70 Ma [[16](#_ENREF_16)]. *Archaeus oblongus* does not represent a minimum age estimate for total-group Carangiformes, as this is defined by earlier occurrences of the unnamed clade containing xiphioids and menids (see below and calibration 5). However, it is the oldest fossil representative that can be assigned to the carangiform total group, and as such represents a first older outgroup to calibrations based on carangiform taxa from Bolca (calibrations 7, 9).

**Total-group Pleuronectiformes, crownward of *Heteronectes***

**Fossil taxon and specimen.** *Amphistium paradoxum*, MNHN BOL 412, Museum national d’Histoire Naturelle, Paris.

**Minimum age.** 49 Ma.

**Phylogenetic justification.** Friedman [[30](#_ENREF_30)] resolved *Amphistium* as the immediate sister taxon of crown Pleuronectiformes in a parsimony analysis of 58 morphological characters.

**Age justification.** *Amphistium* is known from the Pesciara locality of Bolca, Italy. A detailed review of the geology and age of this deposit is given by Papazzoni and colleagues [[13](#_ENREF_13)], but key details are summarized here. Pesciara can be constrained to the narrow interval of overlap between NP14 and SBZ11. This constrains the deposits to no younger than 49 Ma [[14](#_ENREF_14)], which we apply as a minimum age here.

**Notes.** *Amphistium* represents a first stratigraphically consistent outgroup to crown-group pleuronectiforms. The earliest modern flatfishes (*Eobothus*; calibration 11), along with two successive outgroups (*Amphistium*, *Heteronectes*), first appear at Bolca. This sequence informs prior distributions for calibrations within the flatfish crown.

**References**

1. Friedman M., Keck B.P., Dornburg A., Eytan R.I., Martin C.H., Hulsey C.D., Wainwright P.C., Near T.J. 2013 Molecular and fossil evidence place the origin of cichlid fishes long after Gondwanan rifting. *Proceedings of the Royal Society B* **280**, 20131733.

2. Dornburg A., Friedman M., Near T.J. 2015 Phylogenetic analysis of molecular and morphological data highlights uncertainty in the relationships of fossil and living species of Elopomorpha (Actinopterygii: Teleostei). *Molecular Phylogenetics and Evolution* **XX**, xxx-xxx.

3. Benton M.J.D., P. C. J., Asher R.J., Friedman M., Near T.J., Vinther J. 2015 Contraints on the timescale of animal evolutionary history. *Palaeontologia Electronica* **18.1.1FC**, 1-107.

4. Ogg J.G. 2012 Triassic. In *The Geological Time Scale 2012* (eds. Gradstein F.M., Ogg J.G., Schmitz M.D., Ogg G.M.), pp. 681-730. Amsterdam, Elsevier.

5. Friedman M., Keck B.P., Dornburg A., Eytan R.I., Martin C.H., Hulsey C.D., Wainwright P.C., Near T.J. 2013 Molecular and fossil evidence place the origin of cichlid fishes long after Gondwanan rifting. *Proceedings of the Royal Society B: Biological Sciences* **280**(1770).

6. Parham J.F., Donoghue P.C.J., Bell C.J., Calway T.D., Head J.J., Holroyd P.A., Inoue J.G., Irmis R.B., Joyce W.G., Ksepka D.T., et al. 2012 Best practices for justifying fossil calibrations. *Systematic Biology* **61**, 346-359.

7. Davesne D., Friedman M., Barriel V., Lecointre G., Janvier P., Gallut C., Otero O. 2014 Early fossils illuminate character evolution and interrelationships of Lampridiformes (Teleostei, Acanthomorpha). *Zoological Journal of the Linnean Society* **172**, 475-498.

8. Ogg J.G., Hinnov L.A., Huang C. 2012 Cretaceous. In *The Geological Time Scale 2012* (eds. Gradstein F.M., Ogg J.G., Schmitz M.D., Ogg G.M.), pp. 793-853. Amsterdam, Elsevier.

9. Zehren S.J. 1979 The comparative osteology and phylogeny of the Beryciformes (Pisces, Teleostei). *Evolutionary Monographs* **1**, 1-389.

10. Patterson C. 1993 An overview of the early fossil record of acanthomorphs. *Bulletin of Marine Science* **52**, 29-59.

11. Near T.J., Eytan R.I., Dornburg A., Kuhn K.L., Moore J.A., Davis M.P., Wainwright P.C., Friedman M., Smith W.L. 2012 Resolution of ray-finned fish phylogeny and timing of diversification. *Proceedings of the National Academy of Sciences of the USA* **109**, 13698-13703.

12. Otero O. 2004 Anatomy, systematics and phylogeny of both Recend and fossil latid fishes (Teleostei, Perciformes, Latidae). *Zoological Journal of the Linnean Society* **141**, 81-133.

13. Papazzoni C.A., Carnevale G., Fornaciari E., Giusberti L., Trevisani E. 2014 The Pesciara-Monte Postale *Fossil-Lagerstätte*: 1. Biostratography, sedimentology and despositional model. *Reconditi della Società Paleontologica Italiana* **4**, 29-36.

14. Vandenberghe N., Hilgen F.J., Speijer R.P., Ogg J.G., Gradstein F.M., Hammer O., Hollis C.J., Hooker J.J. 2012 The Paleogene Period. In *The Geologic Timescale 2012* (eds. Gradstein F.M., Ogg J.G., Schmitz M.D., Ogg G.M.), pp. 855-921. Amsterdam, Elsevier.

15. Friedman M., Johnson G.D. 2005 A new species of *Mene* (Perciformes: Menidae) from the Paleocene of South America, with notes on paleoenvironment and a brief review of menid fishes. *Journal of Vertebrate Paleontology* **25**, 770-783.

16. Anthonissen D.E., Ogg J.G. 2012 Cenozoic and Cretaceous biochronology of planktonic foraminifera and calcareous nannofossils. In *The Geological Time Scale 2012* (eds. Gradstein F.M., Ogg J.G., Schmitz M.D., Ogg G.M.), pp. 1083-1127. Amsterdam, Elsevier.

17. Bonde N., Andersen S., Hald N., Jakobsen S.L. 2008 *Danekrae--Danmarks Bedste Fossiler*. Copenhagen, Gyldendal; 224 p.

18. Bannikov A.F. 2010 *Fossil Acanthopterygian FIshes (Teleostei, Acanthopterygii)*. Moscow, GEOS; 244 p.

19. Bannikov A.F., Parin N.N. 1997 The list of marine fishes from Cenozoic (upper Paleocene-middle Miocene) localities in southern European Russia and adjacent countries. *Journal of Ichthyology* **37**, 133-146.

20. Friedman M., Johanson Z., Harrington R.C., Near T.J., Graham M.R. 2014 On fossils, phylogenies and sequences of evolutionary change. *Proceedings of the Royal Society B* **281**, 20140115.

21. Friedman M., Johanson Z., Harrington R.C., Near T.J., Harrington R.C. 2013 An early fossil remora (Echeneoidea) reveals the evolutionary assembly of the adhesion disc. *Proceedings of the Royal Society B* **280**, 20131200.

22. O'Toole B. 2002 Phylogeny of the species of the superfamily Echeneoidea (Perciformes: Carangoidei: Echeneidae, Rachycentridae, and Coryphaenidae), with an interpretation of echeneid hitchiking behaviour. *Canadian Journal of Zoology* **80**, 596-623.

23. Sakamoto K., Uyeno T., Micklich N. 2004 *Oligopleuronectes germanicus* gen. et sp. nov., an Oligocene pleuronectid flatfish from Fauenweiler, S-Germany. *Bulletin of the National Science Museum, Tokyo, Series C* **30**, 89-94.

24. Furrer H., Leu U.B. 1998 *Der Landesplattenberg Engi: Forschungsgeschichte, Fossilen und Geologie*. Engi, Siftung Landesplattenberg Engi; 131 p.

25. Smith-Vaniz W.F. 1984 Carangidae: relationships. In *Ontogeny and Systematics of Fishes* (eds. Moser H.G., Richards W.J., Cohen D.M., Fahay M.P., Kendall A.W., Jr., Richardson S.L.), pp. 522-530. Lawrence, Kansas, Allen Press.

26. Gushiken S. 1988 Phylogenetic relationships of the perciform genera of the family Carangidae. *Japanese Journal of Ichthyology* **34**, 443-461.

27. Bannikov A.F. 1990 Fossil carangids and apolectids of the USSR. *Trudy Paleontologicheskogo Instituta* **244**, 1-106.

28. Rögl V.F. 1998 Palaeogeographic considerations for Mediterranean and Paratethys seaways (Oligocene to Miocene). *Annalen des Naturhistorischen Museum in Wien* **99 A**(279-310).

29. Hilgen F.J., Lourens L.J., Van Dam J.A., Beu A.G., Boyes A.F., Cooper R.A., Krijgsman W., Ogg J.G., Piller W.E., Wilson D.S. 2012 The Neogene period. In *The Geologic Timescale 2012* (eds. Gradstein F.M., Ogg J.G., Schmitz M.D., Ogg G.M.), pp. 923-1010. Amsterdam, Elsevier.

30. Friedman M. 2008 The evolutionary origin of flatfish asymmetry. *Nature* **454**(7201), 209-212. (doi:10.1038/nature07108).

31. Chanet B. 1999 Supposed and true flatfishes [Teleostei: Pleuronectiformes] from the Eocene of Monte Bolca, Italy. *Studi e Ricerche sui giaciamenti terziari di Bolca* **8**, 220-243.

32. Hoshino K. 2001 Monophyly of the Citharidae (Pleuronectoidei: Pleuronectiformes: Teleostei) with considerations of pleuronectoid phylogeny. *Ichthyological Research* **48**, 391-404.

33. Chapleau F. 1993 Pleuronectiform relationships: a cladistic reassessment. *Bulletin of Marine Science* **52**, 516-540.

34. Chanet B. 1994 *Eubuglossus eocenicus* (Woodward, 1910) from the upper Lutetian of Egypt, one of the oldest soleids (Teleostei, Pleuronectiformes). *Neues Jahrbuch für Geologie und Paläontologie Abhandlungen* **1994**, 391-398.

35. Chapleau F., Keast A. 1988 A phylogenetic reassessment of the monophyletic status of the family Soleidae, with comments on the suborder Soleoidei (Pisces, Pleuronectiformes). *Canadian Journal of Zoology* **66**, 2797-2810.

36. Gingerich P.D. 1992 Marine mammals (Ceteacea and Sirenia) from the Eocene of Gebel Mokkatam and Fayum, Egypt: stratigraphy, age and paleoenvironments. *University of Michigan Papers on Paleontology* **30**, 1-84.

37. Woodward A.S. 1910 On a fossil sole and fossil eel from the Eocene of Egypt. *Geological Magazine (5)* **7**, 402-404.

38. Baicu D.S., Chanet B. 2002 Les poissons plats fossiles (Teleostei: Pleuronectiformes) de l'Oligocène de Piatra Neamt (Roumanie). *Oryctos* **4**, 17-38.

39. Cooper J.A., Chapleau F. 1998 Monophyly and intrarelationships of the family Pleuronectidae (Pleuronectiformes), with a revised classification. *Fishery Bulletin* **96**, 686-726.

40. Hensley D.A. 1977 Larval development of *Engyophrys senta* (Bothidae), with comments on intermuscular bones in flatfishes. *Bulletin of Marine Science* **27**, 681-703.

41. Carnevale G., Bannikov A.F., Landini W., Sorbini C. 2006 Volhynian (early Sarmatian sensu lato) fishes from Tsurevsky, north Caucasus, Russia. *Journal of Paleontology* **80**, 684-699.

42. Chanet B., Sorbini C. 2001 A male fish *Bothus podas* (Delaroche, 1809) [Pleuronectiformes, Bothidae] in teh Pliocene of the Marecchia River (Italy). *Bollettino della Societá Paleontologica Italiana* **40**, 345-350.

43. Kojumdgieva E.I., Paramonova N.P., Belokrys L.S., Muskhelishvili L.V. 1989 Ecostratigraphic subdivision of the Sarmatian after mollucs. *Geologica Carpathica* **40**, 81-84.

44. Paramonova N.P., Ananova E.N., Andeeva-Grigorovic A.S., Belokrys L.S., Gabunia L.K., Grusinskaja K.F., Kondkarian S.O., Karmishina G.I., Kozirenco T.F., Majsuradze L.S., et al. 1979 Paleontological characteristics of the Sarmatian s.l. and Maeotian of the Ponto-Caspian area and possibilities of correlation to the Sarmatian s.str. and Pannonian of central Paratethys. *Annales Géologiques des Pays Helléniques* **1979**, 961-971.

45. Gavriolv Y.O., Scherbinina E.A., Oberhansli H. 2003 Paleocene-Eocene boundary events in the northeaster Peri-Tethys. *Geological Society of America Special Paper* **369**, 147-168.

46. Schioler P., Andsbjerg J., Clausen O.R., Dam G., Dybkjaer K., Hamberg L., Heilmann-Clausen C., Johannessen E.P., Kristensen L.E., Prince I., et al. 2007 Lithostratigraphy of the Palaeogene-lower Neogene succession of the Danish North Sea. *Geological Survey of Denmark and Greenland Bulletin* **12**, 1-77.
